# Hybrid Inhibitors of DNA Gyrase A and B: Design, Synthesis and Evaluation

**DOI:** 10.3390/pharmaceutics13010006

**Published:** 2020-12-22

**Authors:** Martina Durcik, Žiga Skok, Janez Ilaš, Nace Zidar, Anamarija Zega, Petra Éva Szili, Gábor Draskovits, Tamás Révész, Danijel Kikelj, Akos Nyerges, Csaba Pál, Lucija Peterlin Mašič, Tihomir Tomašič

**Affiliations:** 1 University of Ljubljana, Faculty of Pharmacy, Aškerčeva cesta 7, 1000 Ljubljana, Slovenia; martina.durcik@ffa.uni-lj.si (M.D.); ziga.skok@ffa.uni-lj.si (Ž.S.); janez.ilas@ffa.uni-lj.si (J.I.); nace.zidar@ffa.uni-lj.si (N.Z.); anamarija.zega@ffa.uni-lj.si (A.Z.); danijel.kikelj@ffa.uni-lj.si (D.K.); 2 Synthetic and Systems Biology Unit, Institute of Biochemistry, Biological Research Centre, H-6726 Szeged, Hungary; szilipetraeva@gmail.com (P.É.S.); dras.gabor@gmail.com (G.D.); tamas.revesz.1@gmail.com (T.R.); nyerges.akos@brc.hu (A.N.); cpal@brc.hu (C.P.); 3 Department of Genetics, Harvard Medical School, Boston, MA 02215, USA

**Keywords:** antibacterial, ciprofloxacin, DNA gyrase, dual inhibitor, hybrid

## Abstract

The discovery of multi-targeting ligands of bacterial enzymes is an important strategy to combat rapidly spreading antimicrobial resistance. Bacterial DNA gyrase and topoisomerase IV are validated targets for the development of antibiotics. They can be inhibited at their catalytic sites or at their ATP binding sites. Here we present the design of new hybrids between the catalytic inhibitor ciprofloxacin and ATP-competitive inhibitors that show low nanomolar inhibition of DNA gyrase and antibacterial activity against Gram-negative pathogens. The most potent hybrid **3a** has MICs of 0.5 µg/mL against *Klebsiella pneumoniae*, 4 µg/mL against *Enterobacter cloacae*, and 2 µg/mL against *Escherichia coli*. In addition, inhibition of mutant *E. coli* strains shows that these hybrid inhibitors interact with both subunits of DNA gyrase (GyrA, GyrB), and that binding to both of these sites contributes to their antibacterial activity.

## 1. Introduction

Bacterial resistance poses a major threat to global health, so new therapies against bacterial infections are urgently needed. One of the approaches to address this problem is to target multiple bacterial macromolecules [1]. Among the enzymes that enable multitargeting are the bacterial type IIA topoisomerases: DNA gyrase and topoisomerase IV. These enzymes are well-established targets for antibacterial drug discovery and they have important roles in DNA replication, transcription, repair, and recombination, through altering DNA topology during these processes [2]. They are homologous enzymes, where DNA gyrase consists of two GyrA subunits plus two GyrB subunits, and topoisomerase IV consists of two ParC subunits plus two ParE subunits, thus forming the heterotetrameric A_2_B_2_ and C_2_E_2_ complexes, respectively. The GyrA/ParC subunits contain catalytic sites that bind to DNA, while the GyrB/ParE subunits contain ATP binding sites, and provide the energy required for the catalytic reaction through ATP hydrolysis [3,4]. The fluoroquinolones are catalytic site inhibitors that have been successfully used in clinical applications since the introduction of nalidixic acid, and they remain an important class of antibacterial agents for the treatment of Gram-positive and Gram-negative bacterial infections [5,6]. On the other hand, the only inhibitor of GyrB that interacts with the ATP binding site that reached the clinic was novobiocin, although therapeutic use was discontinued due to toxicity and the emergence of target-based resistance [7].

Mutations conferring resistance to novobiocin in *Escherichia coli* GyrB are most commonly found in the Arg136 residue, although other residues have also been identified where their mutation can lead to novobiocin resistance (Asp73, Gly77, Ile78, Thr165) [8]. Fluoroquinolones such as ciprofloxacin, levofloxacin, and moxifloxacin are generally effective against infections caused by Gram-positive and Gram-negative pathogens, and hence they are widely used in medicine. However, resistance to these agents has emerged and continues to spread despite recommendations to limit their use [9,10]. Indeed, resistance to fluoroquinolones has been detected for most bacterial infections treated with these antibiotics. In particular, the difficult to treat infections here include: (i) urinary tract infections caused by *E. coli*; (ii) respiratory infections caused by *Streptococcus pneumoniae*, which is a leading cause of community-acquired pneumonia; (iii) intra-abdominal infections caused by resistant *E. coli*, *Salmonella* spp., and *Shigella*; (iv) infections of the skin and skin structures through methicillin-resistant *Staphylococcus aureus*, where fluoroquinolones resistance is spread worldwide; and (v) gonococcal infections through *Neisseria gonorrhoeae*, where ciprofloxacin resistance already appeared in the late 1990s [11].

The mechanisms behind this fluoroquinolones resistance include: (i) chromosomal mutations that cause increased antibiotic efflux or reduced uptake, and hence reduced intracellular accumulation; (ii) plasmid-acquired genes that encode drug-modifying enzymes, efflux pumps, or target protection proteins; and (iii) most commonly and clinically significant, gene mutations at the target site [5,12]. Mutations can occur within the quinolone-resistance-determining regions of GyrA and/or ParC. The most common mutation sites in *E. coli* GyrA are Ser83 and Asp87, which are the key amino-acid residues for fluoroquinolone binding, and the corresponding homologous positions in ParC, as Ser80 and Glu84 [11,13].

For these reasons, there is an urgent need for development of new drugs that circumvent common fluoroquinolone resistance mechanisms. Due to the structural similarities between DNA gyrase and topoisomerase IV, dual-targeting inhibitors can be developed that simultaneously inhibit GyrA and ParC or GyrB and ParE. The development of bacterial target-based resistance to such inhibitors will be less likely because resistance-conferring mutations would need to occur simultaneously at both targets, which is unlikely to happen [1,7,14]. In the present study, we used a different approach to design dual-targeting antimicrobial compounds. Specifically, we aimed to inhibit both the catalytic and the ATP binding sites of the same target protein.

**Figure 1:**
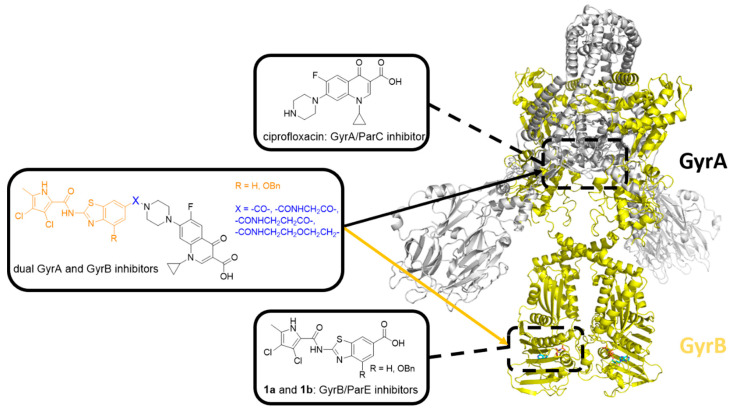
Structure of *E. coli* DNA gyrase (Protein Data Bank (PDB) entry: 6RKW [23]). The two GyrA subunits are in gray, and the binding site of GyrA/ParC inhibitor ciprofloxacin is represented by the upper black dashed rectangle. The two GyrB subunits are in yellow, and the binding site of GyrB/ParE inhibitors **1a** and **1b** is represented by the lower black dashed rectangle. The general structure of GyrA/ParC and GyrB/ParE inhibitor hybrids is shown in the middle.

In recent years, we have investigated and reported on several structural types of ATP-competitive GyrB/ParE inhibitors [15,16,17,18,19]. Recently, we also reported the discovery of GyrB inhibitor/ ciprofloxacin hybrids [20]. These previously designed hybrids showed weak antibacterial activities, which were shown to be mainly due to interactions with the GyrA and/or ParC subunits. To overcome this difficulty, we focused on our recently developed balanced dual GyrB/ParE ATP-competitive inhibitors **1a** and **1b** (Figure 1). These inhibitors have potent antibacterial activity against several Gram-positive and Gram-negative bacterial strains [21]. In this paper, we present new hybrids between ciprofloxacin and **1a** or **1b**. By combining these molecules, we have reached superior antibacterial activities due to the interactions of these hybrids with both subunits, as GyrA and GyrB or ParC and ParE. Importantly, the compounds are effective against Gram-negative strains of bacteria that belong to the group of resistant ‘ESKAPE’ pathogens that pose a major threat to society and health (i.e., *Enterococcus faecium*, *Staphylococcus aureus*, *Klebsiella pneumoniae*, *Acinetobacter baumannii*, *Pseudomonas aeruginosa*, *Enterobacter* spp.) [22].

## 2. Materials and Methods

### 2.1. General Information—Chemistry

Chemicals were obtained from Acros Organics (Geel, Belgium), Sigma-Aldrich (St. Louis, MO, USA), and Apollo Scientific (Stockport, UK), and were used without further purification. Analytical thin-layer chromatography was performed on silica gel plates (Merck 60 F254; 0.25 mm), with visualization with UV light (at 254 nm and 366 nm) and spray reagent ninhydrin. Column chromatography was carried out on silica gel 60 (particle size, 240–400 mesh). Analytical reversed-phase HPLC analysis was performed on a liquid chromatography system (1260 Infinity II LC; Agilent Technologies Inc., Santa Clara, CA, USA). A C18 column was used (3.5 μm, 4.6 mm × 150 mm; XBridge; Waters, Milford, MA, USA), with a flow rate of 1.5 mL/min and a sample injection volume of 10 µL. The mobile phase consisted of acetonitrile (solvent A) and 0.1% formic acid in 1% acetonitrile in ultrapure water (solvent B). The gradient (defined for solvent A) was: 0–1.0 min, 25%; 1.0–6.0 min, 25–98%; 6.0–6.5 min, 98%; 6.5–7.5 min, 98–25%; 7.5–10.5 min, 25%. Ultrapure water was obtained with a Milli-Q Advantage A10 water purification system (Millipore, Merck, Burlington, MA, USA). Melting points were determined on a hot stage microscope (Reichert) and are uncorrected. ^1^H NMR spectra were recorded at 400 MHz (Bruker AVANCE III 400 spectrometer; Bruker Corporation, Billerica, MA, USA) in DMSO-*d*_6_ solutions, with tetramethylsilane as the internal standard. Infrared (IR) spectra were recorded (Thermo Nicolet Nexus 470 ESP FT-IR spectrometer; Thermo Fisher Scientific, Waltham, MA, USA). Mass spectra were obtained using a compact mass spectrometer (Advion expression; Advion Inc., Ithaca, NY, USA). High-resolution mass spectrometry was also performed (Exactive Plus Orbitrap; Thermo Fisher Scientific, Waltham, MA, USA). Detailed synthetic procedures and analytical data for all of the compounds are in Appendix A. ^1^H NMR spectra and HPLC chromatograms can be found in Supplementary Materials.

### 2.2. Molecular Docking

Molecular docking calculations were performed using Schrödinger Release 2020-1 (Schrödinger, LLC, New York, NY, USA, 2020). The crystal structures of *S. aureus* DNA gyrase A in complex with moxifloxacin (PDB entry: 5CDQ [24]) and *S. aureus* DNA gyrase B in complex with **1b** (PDB entry: 6TCK [21]) were retrieved from the Protein Data Bank. The proteins were prepared using Protein Preparation Wizard, with the default settings. The receptor grid was calculated for the ligand-binding site, and the designed hybrids were docked using the Glide XP protocol, as implemented in Schrödinger Release 2020-1 (Glide, Schrödinger, LLC, New York, NY, USA, 2020). Figures were prepared with PyMOL [25].

### 2.3. Determination of Inhibitory Activities on Escherichia coli DNA Gyrase and Topoisomerase IV

The assay for determination of the IC_50_ values was performed according to previously reported procedures [26].

Briefly, inhibitory activities were determined using supercoiling (for DNA gyrase) and relaxation (for topoisomerase IV) assay kits (Inspiralis, Norwich, UK) on streptavidin-coated 96-well microtiter plates from Thermo Scientific Pierce (Thermo Fisher Scientific, Waltham, MA, USA). First, the plates were rehydrated with Wash Buffer and the biotinylated oligonucleotide TFO1 was then immobilized. After washing off the unbound oligonucleotide with Wash Buffer, the enzyme assay was performed. Into each well was added 24 µL of mixture containing 6 µL of Assay Buffer (containing ATP), 0.75 µL of relaxed (for *E. coli* DNA gyrase supercoiling assay) or supercoiled (for *E. coli* topoisomerase IV relaxation assay) pNO1 plasmid and 17.25 µL of water. Additionally, 3 µL of a solution of the inhibitor in 10% DMSO containing 0.008% Tween 20 and 3 µL of enzyme (1.5 U) in Dilution Buffer were also added to the wells. Reaction solutions were incubated at 37 °C for 30 min. The TF buffer was added to terminate the enzymatic reaction and after additional incubation for 30 min at room temperature, which allowed for triplex formation (biotin–oligonucleotide–plasmid), the unbound plasmid was washed off using TF buffer. Promega Diamond dye in T10 buffer was then added. After another 15 min incubation at room temperature in the dark, the fluorescence (excitation: 485 nm, emission: 535 nm) was measured with an automated microplate reader (Synergy^TM^ H4, BioTek, Winooski, VT, USA). Initial screening was done at 10 µM and 1 µM concentrations of inhibitors and for the active inhibitors at these concentrations, IC_50_ values were determined using seven concentrations of tested compounds. The test concentration range in *E. coli* DNA gyrase assay was 0.028–10 µM for compounds **3a** and **3b**, 0.0028–1 µM for **7a**, **7b**, and **11a**, and 0.0011–0.4 for **11b**. Compounds were diluted using 0.375-fold serial dilution steps of the given compound. The test concentration range in *E. coli* topoisomerase IV assay was 0.063–4 µM for compound **3a** and 0.156–10 µM for compounds **7a**, **7b**, and **11b**. Here, the compounds were diluted using 2-fold serial dilution steps of the given compound. GraphPad Prism 6.0 software was used to calculate the IC_50_ values, which were determined in at least two independent measurements, and their means are given as the final result. Novobiocin was used as the positive control. Dose-response curves can be found in Supplementary Materials.

### 2.4. Determination of Antibacterial Activities

The following clinical microbiology control strains were obtained from American Type Culture Collection (ATCC) via Microbiologics Inc. (St. Cloud, MN, USA): *A. baumannii* (ATCC 17978); *E. coli* (ATCC 25922); *K. pneumoniae* (ATCC 10031); *P. aeruginosa* (ATCC 27853); and *Enterobacter cloacae* spp. *cloacae* (ATCC 13047). *E. coli* MG1655 originated from the laboratory collection of Dr. Csaba Pál. The GyrA and GyrB mutant strains of *E. coli* MG1655 were constructed using pORTMAGE [27] recombineering (Addgene plasmid #120418; http://n2t.net/addgene:120418; RRID:Addgene_120418), according to the published protocol [28]. *E. coli* K-12 BW25113 single-gene knockout mutant lines ΔdapF, ΔmrcB, ΔsurA, ΔacrB and ΔtolC originated form the Keio collection copy owned by the laboratory of Dr. Csaba Pál [29].

Cation-adjusted Mueller Hinton II broth (MHBII) was used for growth of the bacteria under standard laboratory conditions, for antimicrobial susceptibility tests, and for selection of resistant variants. To prepare the MHBII broth, 22 g MHBII powder (containing 3 g beef extract, 17.5 g acid hydrolysate of casein, 1.5 g starch; Becton, Dickinson and Co., Franklin Lakes, NJ, USA) was dissolved in 1 L water. MHBII agar was prepared by addition of 14 g agar (Bacto; Molar Chemicals, Halásztelek, Hungary) to 1 L broth.

Minimum inhibitory concentrations (MICs) were determined using a standard serial broth microdilution technique, according to the Clinical and Laboratory Standards Institute guidelines [30]. Bacterial strains were inoculated onto MHBII agar plates and grown overnight at 37 °C. Next, three individual colonies from each strain were inoculated into 1 mL MHBII medium and propagated at 37 °C overnight, with agitation at 250 rpm. For *Enterococcus* sp., the cells were plated in BHI agar plates, and BHI broth was used to determine the MICs. To perform the MIC assays, 12-step serial dilutions using two-fold dilution steps of the given compound (each dissolved in 100% DMSO) were generated in 96-well microtiter plates (Corning Inc., Corning, NY, USA). The concentrations used were: 64, 32, 16, 8, 4, 2, 1, 0.5, 0.25, 0.125, 0.0625, and 0.03125 µg/mL. Following the dilutions, each well was seeded with 5 × 10^4^ bacterial cells. Each measurement was performed in three parallel replicates, and to avoid possible edge effects in the microwell plates, the outside rows (A, H) were filled with sterile medium. Following the inoculations, the plates were covered with the lids and wrapped in polyethylene plastic bags, to minimize evaporation but to allow O_2_ transfer. The plates were incubated at 37 °C under continuous shaking at 150 rpm for 18 h. After incubation, the OD_600_ of each well was measured using a microplate reader (Synergy 2; Biotek, Winooski, VT, USA). The MICs were defined as the antibiotic concentrations which inhibited the growth of the bacterial cultures; i.e., the drug concentration where the average OD_600_ increment of the three technical replicates was below 1.5-fold the background OD increment.

## 3. Results and Discussion

### 3.1. Design

**Figure 2:**
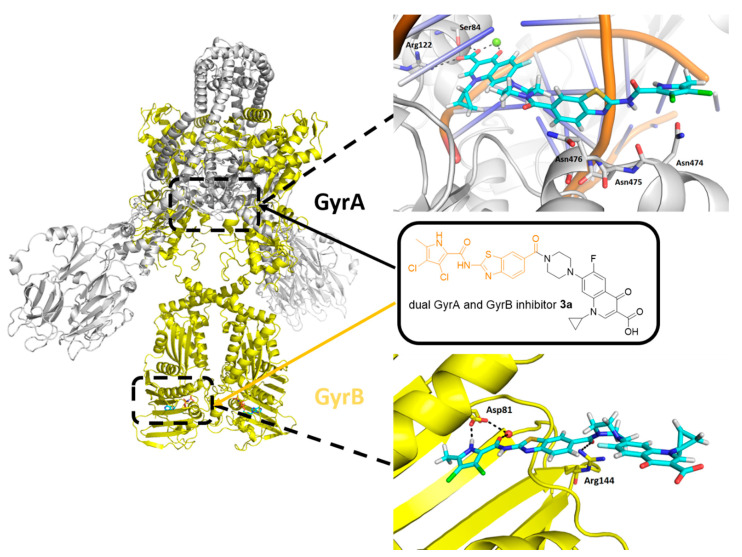
Docking binding modes of the representative hybrid **3a** (cyan sticks) in the catalytic site of GyrA (*S. aureus* GyrA in gray; DNA in orange; PDB entry: 5CDQ [24]) and in the ATP-binding site of GyrB (*S. aureus* GyrB in yellow; PDB entry: 6TCK [21]). For clarity, only amino-acid residues forming hydrogen bonds (dashed lines) are presented as sticks. The magnesium ion in GyrA is a green sphere, while the structural water in GyrB is a red sphere.

Our first series of dual GyrA and GyrB inhibitor hybrids was designed by combining the GyrA inhibitor ciprofloxacin with benzothiazole-based GyrB inhibitors [20]. Although the GyrB inhibitors used in the first series of hybrids showed potent DNA gyrase inhibition, they showed only low antibacterial activity [17]. When the hybrids were tested against bacteria, we showed that in the bacteria they only bound to GyrA, and not to GyrB, which means that the observed antibacterial activity was mainly due to the ciprofloxacin part that interacted with GyrA. In the present series, we combined our balanced dual-targeting GyrB and ParE inhibitors **1a** and **1b** with potent antibacterial activity against Gram-positive and Gram-negative strains with ciprofloxacin [21]. The new hybrids were prepared either by direct fusion of inhibitors **1a** and **1b** with ciprofloxacin, or by linking the two molecules with linkers of different lengths (i.e., glycine in **7a**, β-alanine in **7b** or 2-ethoxyethyl in **11a** and **11b**). The design of the new dual GyrA and GyrB inhibitors is shown in Figure 1 and Figure 2.

Molecular docking showed that all designed hybrids **3a**, **3b**, **7a**, **7b**, **11a** and **11b** can bind to either GyrA or GyrB. As an example, the docking binding mode of a representative hybrid **3a** in GyrA and GyrB active sites is presented in Figure 2. Docking of **3a** to the catalytic site of GyrA reproduced the binding conformation of the fluoroquinolone portion, as observed for moxifloxacin in the crystal structure. Important hydrogen bonds were formed with Ser84, Arg122 and a magnesium ion, while π-stacking interactions were formed with DNA bases. The GyrB part of hybrid **3a** made additional hydrophobic contacts with Asn474–476 (Figure 2). At the ATP binding site of GyrB, the pyrrolamide moiety of **3a** interacted with Asp81 and a structural water molecule, as observed for **1b** in the crystal structure. The carbonyl group connecting the benzothiazole moiety to the piperidine nitrogen atom of ciprofloxacin formed a hydrogen bond with Arg144. Furthermore, the ciprofloxacin part of **3a** pointed towards the bulk solvent and did not interfere with the binding (Figure 2). Similar binding modes were obtained also for other designed GyrA/GyrB inhibitor hybrids.

### 3.2. Chemistry

**Scheme 1:**
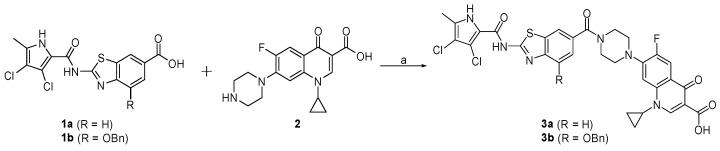
Reagents and conditions: (**a**) 1-ethyl-3-(3-dimethylaminopropyl)carbodiimide (EDC), 1-hydroxybenzotriazole (HOBt), *N*-methylmorpholine (NMM), *N*,*N*-dimethylformamide (DMF), rt, 15 h.

The fused hybrids **3a** and **3b** were prepared in the one-step synthesis presented in Scheme 1. Compounds **1a** and **1b** [21] were coupled to ciprofloxacin (**2**) using the reagents 1-ethyl-3-(3-dimethylaminopropyl)carbodiimide (EDC) and 1-hydroxybenzotriazole (HOBt), to obtain the final compounds **3a** and **3b**.

**Scheme 2:**
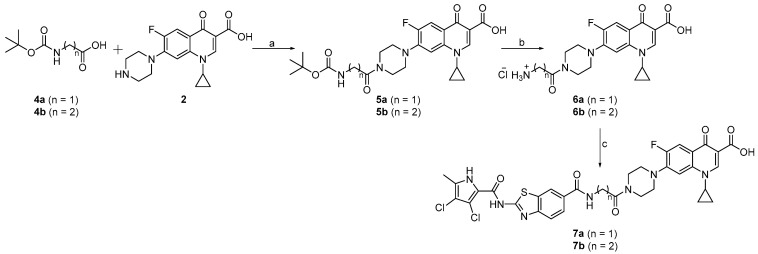
Reagents and conditions: (**a**) EDC, HOBt, NMM, DMF, rt, 15 h; (**b**) 4 M HCl in 1,4-dioxane, 1,4-dioxane, rt, 3 h; (**c**) **1a**, EDC, HOBt, NMM, DMF, rt, 15 h.

The synthesis of the linked compounds **7a** and **7b** is shown in Scheme 2. First, ciprofloxacin (**2**) was coupled to *N*-(*tert*-butoxycarbonyl)glycine (**4a**) or 3-(*N*-(*tert*-butoxycarbonyl)amino)propanoic acid (**4b**) using EDC- and HOBt-promoted coupling, to obtain compounds **5a** and **5b**. The Boc protecting groups of **5a** and **5b** were removed by acidolysis, and the obtained intermediates **6a** and **6b** were coupled to **1a** to obtain the desired compounds **7a** and **7b**.

**Scheme 3:**
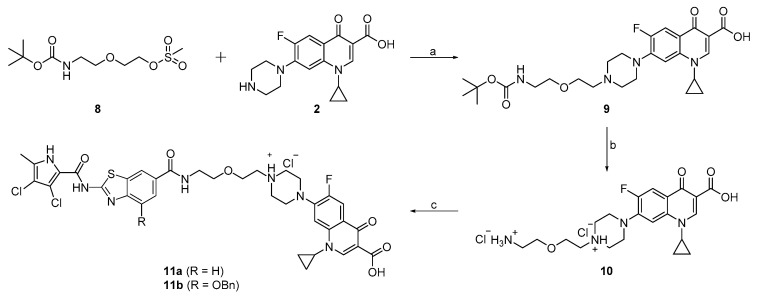
Reagents and conditions: (**a**) Et_3_N, DMF/H_2_O, 80 °C, 72 h; (**b**) 4 M HCl in 1,4-dioxane, 1,4-dioxane, rt, 3 h; (**c**) **1a** or **1b**, EDC, HOBt, NMM, DMF, rt, 15 h.

The compounds **11a** and **11b** were synthesized according to Scheme 3. The reaction between 2-(2-((*tert*-butoxycarbonyl)amino)ethoxy)ethyl methanesulfonate (**8**) and ciprofloxacin (**2**) using triethylamine (Et_3_N) in DMF and water yielded intermediate **9**. The Boc protecting group of **9** was removed with HCl in 1,4-dioxane to obtain compound **10**. In the final step, compound **10** was coupled to **1a** or **1b**, to obtain the final compounds **11a** and **11b**.

### 3.3. Enzyme Inhibition and Antibacterial Activities

Six new hybrids were prepared and tested for their inhibitory activities against DNA gyrase and topoisomerase IV from *E. coli* in supercoiling and relaxation assays, respectively (Table 1). The antibacterial activities of the hybrids were tested against Gram-negative bacteria from the ESKAPE group of pathogens, and are shown in Table 2.

Four hybrids (**7a**, **7b**, **11a, 11b**) showed potent nanomolar inhibitory activities against DNA gyrase from *E. coli* (IC_50_ range, 14–130 nM; Table 1), which were comparable or superior to the activity of the GyrA inhibitor ciprofloxacin (IC_50_, 120 nM; Table 1). Compared to the GyrB inhibitors **1a** and **1b**, the hybrids showed weaker inhibition, with the exception of **11b**, which showed very promising activity, with an IC_50_ of 14 nM. Inhibition of topoisomerase IV was weaker for all of these tested compounds, with low micromolar activities for **3a**, **7a**, **7b**, and **11b**, and lack of activity for **3b** and **11a**. Ciprofloxacin also inhibited topoisomerase IV in the low micromolar range, with an IC_50_ of 5.4 µM. These data demonstrate that the addition of these linkers that give flexibility to the molecules was beneficial for the inhibitory activities: here, the fused molecules **3a** and **3b** showed weaker inhibition of DNA gyrase compared to the linked molecules **7a**, **7b**, **11a**, and **11b**. On the contrary, the best antibacterial activity was obtained for the fused hybrid **3a**. Compound **3a** showed potent MICs against *K. pneumoniae* (0.5 µg/mL), *E. cloacae* (4 µg/mL), and *E. coli* (2 µg/mL) (Table 2). Compared to the other five hybrids, **3a** is the smallest molecule, so one hypothesis for its superior activity is that this size difference contributes to better cellular uptake. However, because of experiments detailed below we came to doubt that differences in cellular uptake account for its superior activity. Compounds **7a** and **7b**, with the glycine and β-alanine linkers, showed no significant antibacterial activities, except against *K. pneumoniae*, while the ether compounds **11a** and **11b** were active against *E. coli* in addition to *K. pneumoniae* (Table 2). The activities of **11a** and **11b** against these two bacterial strains were comparable to the GyrB inhibitors **1a** and **1b**, while they were lower than the antibacterial activity of ciprofloxacin. In addition, the dual-targeting ligands **3a** and **11b** showed promising antibacterial activities also against *E. cloacae* (MICs, 4, 16 µg/mL, respectively), against which the GyrB inhibitors **1a** and **1b** were inactive. Compound **3b** showed only weak micromolar enzyme inhibition, and was therefore almost inactive against all of these bacterial strains.

To study the factors that influence the antibacterial activities of these compounds, the hybrids were also tested on *E. coli* BW25113 wild-type and mutant strains (Table 3). The first three of the mutated genes for *dapF*, *mrcB*, and *surA* are involved in peptidoglycan biosynthesis and maturation of outer membrane proteins [31]; this leads to mutants with disturbed cell walls, which are thus more permeable. The other two mutants were for the *acrB* and *tolC* genes that encode efflux pump proteins [32]. When the hybrids were tested against the Δ*dapF*, Δ*mrcB*, and Δ*surA* mutants with impaired cell walls, there were no differences in the activities compared to the wild-type strain. Therefore, poor penetration does not appear to be the main reason for the weak activities of these GyrA/GyrB inhibitor hybrids. In contrast, when tested against the Δ*tolC* mutant with an impaired efflux pump, the activities were improved for all of these tested hybrids. For **3a** and **11b**, the MICs showed changes of 4-fold or 8-fold, while for **7a**, **7b**, and **11a** the changes were above 32-fold or 64-fold. From these data, we can conclude that the hybrids **7a**, **7b**, and **11a** underwent strong efflux from the bacterial cell, which might be the reason for their weak activity or inactivity against the wild-type strains (Table 2). Although **3a** and **11b** also showed improved activities here, the MIC changes were not as pronounced, and their good activities on the wild-type strain show that the efflux was not detrimental to their antibacterial activities.

To investigate whether the observed antibacterial activities of these hybrids is due to their interactions with the catalytic or the ATP binding sites of DNA gyrase, or both, we tested them against three *E. coli* MG1655 strains in the presence of an efflux-pump substrate phenylalanine-arginine β-naphthylamide (PAβN): wild-type; a GyrB R136C mutant carrying a mutation at the ATP binding site; and the GyrA S83L, D87N and ParC S80I, E84G mutants, with the fluoroquinolone-binding site mutated (Table 4). These mutated amino acids are the most common sites for target-based resistance in *E. coli*. The results for these mutant strains confirm the hypothesis that these compounds can interact with both subunits; i.e., GyrA (and/or ParC) and GyrB. Compound **7b** showed equipotent antibacterial activities against the strain with a mutated GyrB binding site and against the strain with the mutated fluoroquinolone binding site (GyrA and ParC). The activities against the two mutants were also comparable for compounds **7a** and **11b**, which suggests that **7a**, **7b**, and **11b** have a balanced interaction with both of the binding sites. For **3a** and **11a**, the mutation in the GyrB binding site did not result in weaker activities compared to the wild type, while the mutation at the fluoroquinolone binding site did; this indicated that these two compounds interacted more strongly with the GyrA and ParC subunits. Nevertheless, the activities for this mutant were not completely lost. From these data, it can be concluded that our new hybrids can interact with both the GyrA and GyrB binding sites.

## 4. Conclusions

In summary, we have designed and synthesized new hybrid ligands that can interact with both the catalytic and the ATP binding sites of DNA gyrase. These new hybrids show low nanomolar inhibitory activities on *E. coli* DNA gyrase, and good antibacterial activities, especially against *K. pneumoniae* and *E. coli*, two pathogens from the problematic ESKAPE group. Additionally, compounds **3a** and **11b** do not undergo extensive efflux. Testing of these compounds on mutant *E. coli* strains confirmed that they can bind to both the GyrA (and/or ParC) and GyrB binding sites. Targeting both of the binding sites results in potent antibacterial activities against Gram-negative pathogens, and should also have a strong advantage in terms of delayed or prevented emergence of bacterial resistance.

## Figures and Tables

**Table 1:** Inhibitory activities of the new hybrid compounds against *E. coli* DNA gyrase and topoisomerase IV.

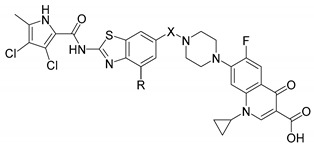				
Cpd.	R	X	IC_50_ [nM] ^1^	
			*E. coli*	
			DNA Gyrase	Topoisomerase IV
**3a**	H	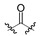	450 ± 180	1000 ± 150
**3b**	OBn	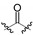	3300 ± 1500	>10,000
**7a**	H	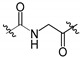	92 ± 10	2500 ± 1300
**7b**	H	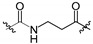	130 ± 10	3100 ± 0
**11a** ^2^	H	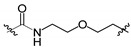	130 ± 30	>10,000
**11b** ^3^	OBn	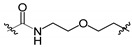	14 ± 9	2500 ± 1100
**1a** ^4^			13 ± 0	500 ± 280
**1b** ^5^			<10	350 ± 50
CP ^6^			120 ± 20	5400 ± 2100
NB ^7^			170 ± 20	11,000 ± 2000

^1^ Concentration of compound (mean ±SD, in nM) that inhibits the enzyme activity by 50%; ^2^, ^3^ Compounds **11a** and **11b** were obtained as hydrochloride salts; ^4^, ^5^ **1a** and **1b**, GyrB and ParE inhibitors; ^6^ CP, ciprofloxacin; ^7^ NB, novobiocin.

**Table 2:** Minimum inhibitory concentration (MIC) values of the new hybrid compounds against the indicated Gram-negative bacterial strains.

Cpd.	MIC [µg/mL] ^1^				
	*A. baumannii* ATCC 17978	*P. aeruginosa* ATCC 27863	*K. pneumoniae* ATCC 10031	*E. cloacae* spp. *cloacae* ATCC 13047	*E. coli* ATCC 25922
**3a**	16	64	0.5	4	2
	(23.4 µM)	(93.6 µM)	(0.73 µM)	(5.85 µM)	(2.93 µM)
**3b**	>64	>64	>64	>64	>64
	(>81.0 µM)	(>81.0 µM)	(>81.0 µM)	(>81.0 µM)	(>81.0 µM)
**7a**	>64	>64	4	>64	>64
	(>86.4 µM)	(>86.4 µM)	(5.40 µM)	(>86.4 µM)	(>86.4 µM)
**7b**	>64	>64	8	>64	>64
	(>84.8 µM)	(>84.8 µM)	(10.6 µM)	(>84.8 µM)	(>84.8 µM)
**11a**	>64	>64	4	64	32
	(>82.9 µM)	(>82.9 µM)	(5.18 µM)	(82.9 µM)	(41.5 µM)
**11b**	64	>64	1	16	4
	(72.9 µM)	(>72.9 µM)	(1.14 µM)	(18.2 µM)	(4.56 µM)
**1a** ^2^	4	8	1	>64	4
	(10.8 µM)	(21.6 µM)	(2.70 µM)	(>173 µM)	(10.8 µM)
**1b** ^3^	2	2	4	>64	16
	(4.20 µM)	(4.20 µM)	(8.40 µM)	(>134 µM)	(33.6 µM)
CP ^4^	0.25	0.25	<0.03125	<0.03125	<0.03125
	(0.75 µM)	(0.75 µM)	(<0.0943 µM)	(<0.0943 µM)	(<0.0943 µM)

^1^ MIC, minimum inhibitory concentration; ^2^, ^3^ **1a** and **1b**, GyrB and ParE inhibitors; ^4^ CP, ciprofloxacin. Measurements were performed according to the Clinical and Laboratory Standards Institute guidelines, with three independent measurements.

**Table 3:** Minimum inhibitory concentrations of these new hybrid compounds against *E. coli* BW25113 wild-type and mutant strains with impaired cell-wall structure (Δ*dapF*, Δ*mrcB*, Δ*surA*) and efflux pumps (Δ*acrB*, Δ*tolC*).

Cpd.	Minimum Inhibitory Concentration [µg/mL]					
	*E. coli* BW25113 WT ^1^	*E. coli* BW25113 Δ*dapF*	*E. coli* BW25113 Δ*mrcB*	*E. coli* BW25113 Δ*surA*	*E. coli* BW25113 Δ*acrB*	*E. coli* BW25113 Δ*tolC*
**3a**	4	4	4	4	4	1
	(5.85 µM)	(5.85 µM)	(5.85 µM)	(5.85 µM)	(5.85 µM)	(1.46 µM)
**3b**	>64	>64	>64	>64	>64	16
	(>81.0 µM)	(>81.0 µM)	(>81.0 µM)	(>81.0 µM)	(>81.0 µM)	(20.3 µM)
**7a**	>64	>64	>64	>64	>64	2
	(>86.4 µM)	(>86.4 µM)	(>86.4 µM)	(>86.4 µM)	(>86.4 µM)	(2.70 µM)
**7b**	>64	>64	>64	>64	>64	2
	(>84.8 µM)	(>84.8 µM)	(>84.8 µM)	(>84.8 µM)	(>84.8 µM)	(2.65 µM)
**11a**	32	32	32	16	32	0.5
	(41.5 µM)	(41.5 µM)	(41.5 µM)	(20.7 µM)	(41.5 µM)	(0.65 µM)
**11b**	8	8	8	8	8	1
	(9.11 µM)	(9.11 µM)	(9.11 µM)	(9.11 µM)	(9.11 µM)	(1.14 µM)
CP ^2^	<0.03125	<0.03125	<0.03125	<0.03125	<0.03125	<0.03125
	(<0.0943 µM)	(<0.0943 µM)	(<0.0943 µM)	(<0.0943 µM)	(<0.0943 µM)	(<0.0943 µM)

^1^ WT, wild-type; ^2^ CP, ciprofloxacin.

**Table 4:** Minimum inhibitory concentrations of these new hybrid compounds against the three indicated *E. coli* MG1655 strains. The assays were carried out in the presence of 50 µg/mL PAβN.

Cpd.	Minimum Inhibitory Concentration [µg/mL]		
	*E. coli* MG1655 WT ^1^	*E. coli* MG1655 GyrB R136C	*E. coli* MG1655 GyrA S83L, D87N ParC S80I, E84G
**3a**	0.5	0.5	16
	(0.73 µM)	(0.73 µM)	(23.4 µM)
**3b**	64	32	>64
	(81.0 µM)	(40.5 µM)	(>81.0 µM)
**7a**	1	2	0.5
	(1.35 µM)	(2.70 µM)	(0.675 µM)
**7b**	1	1	1
	(1.325 µM)	(1.325 µM)	(1.325 µM)
**11a**	0.5	0.5	8
	(0.65 µM)	(0.65 µM)	(10.4 µM)
**11b**	1	2	4
	(1.14 µM)	(2.28 µM)	(4.56 µM)
CP ^2^	<0.03125	<0.03125	64
	(<0.0943 µM)	(<0.0943 µM)	(193 µM)

^1^ WT, wild type; ^2^ CP, ciprofloxacin.

## Data Availability

The data presented in this study are available at www.mdpi.com/xxx/s1.

## Supplementary Materials

The following are available online at https://www.mdpi.com/1999-4923/13/1/6/s1: Figure S1. Dose-response curves for compounds **3a**, **3b**, **7a**, **7b**, **11a**, and **11b** for *E. coli* DNA gyrase, Figure S2. Dose-response curves for compounds **3a**, **7a**, **7b**, and **11b** for *E. coli* topoisomerase IV, Figure S3. ^1^H NMR spectra of compound **3a**, Figure S4. HPLC chromatogram of compound **3a**, Figure S5. ^1^H NMR spectra of compound **3b**, Figure S6. HPLC chromatogram of compound **3b**, Figure S7. ^1^H NMR spectra of compound **7a**, Figure S8. HPLC chromatogram of compound **7a**, Figure S9. ^1^H NMR spectra of compound **7b**, Figure S10. HPLC chromatogram of compound **7b**, Figure S11. ^1^H NMR spectra of compound **11a**, Figure S12. HPLC chromatogram of compound **11a**, Figure S13. ^1^H NMR spectra of compound **11b**, Figure S14. HPLC chromatogram of compound **11b**.

## Appendix A

### Synthetic Procedures and Analytical Data

General procedure A. Synthesis of compounds **3a-b**, **5a-b**, **7a-b**, and 11a-b (with 3a as the example). A solution of 2-(3,4-dichloro-5-methyl-1*H*-pyrrole-2-carboxamido)benzo[*d*]thiazole-6-carboxylic acid (**1a**, 75 mg, 0.203 mmol) in *N*,*N*-dimethylformamide (8 mL) was cooled to 0 °C, and then EDC (47 mg, 0.244 mmol), HOBt (36 mg, 0.264 mmol), and *N*-methylmorpholine (45 µL, 0.406 mmol) were added, and the reaction mixture was stirred for 30 min at 0 °C. Ciprofloxacin (**2**, 67 mg, 0.203 mmol) was then added, and the reaction mixture was stirred at room temperature overnight. The solvent was evaporated in vacuo, with ethyl acetate (30 mL) and 10% citric acid (20 mL) added to the residue. The precipitate obtained was filtered off, washed with ethyl acetate, and dried.

**1-Cyclopropyl-7-(4-(2-(3,4-dichloro-5-methyl-1*H*-pyrrole-2-carboxamido)benzo[*d*]thiazole-6-carbonyl)piperazin-1-yl)-6-fluoro-4-oxo-1,4-dihydroquinoline-3-carboxylic acid (3a)**. Yield: 91 mg (65.7%); dark purple solid; mp > 300 °C. ^1^H NMR (400 MHz, DMSO-*d*_6_) δ [ppm] 1.15–1.23 (m, 2H, cyclopropyl-CH_2_), 1.30–1.37 (m, 2H, cyclopropyl-CH_2_), 2.28 (s, 3H, pyrr-CH_3_), 3.30–3.45 (signal is overlapped with the signal for water, 4H, 2 × piperazine-CH_2_), 3.65–3.90 (m, 5H, 2 × piperazine-CH_2_, CH), 7.58 (dd, 1H, *J* = 8.4, 1.6 Hz, Ar-H′-5), 7.62 (d, 1H, *J* = 7.6 Hz, Ar-H′-4/Ar-H-8), 7.85 (s, 1H, Ar-H′-4/Ar-H-8), 7.96 (d, 1H, *J* = 13.1 Hz, Ar-H-5), 8.19 (s, 1H, Ar-H′-7), 8.69 (s, 1H, Ar-H-2), 11.86 (s, 1H, pyrr-NH/CONH), 12.39 (s, 1H, pyrr-NH/CONH), 15.21 (s, 1H, COOH). IR (ATR) ν [cm^−^¹] = 3368 (NH), 3173 (NH), 3066 (OH), 1713 (CO), 1663 (CO), 1603, 1530, 1491, 1331, 1256, 1162, 1085, 1020, 944, 900, 830, 809, 771, 742, 706, 624, 552, 517. HRMS (ESI^-^) *m/z* for C_31_H_24_Cl_2_FN_6_O_5_S ([M-H]^-^): calculated: 681.0885, found: 681.0909, delta: 3.66 ppm. HPLC: *t*_r_ 6.86 min (95.8% at 254 nm).

**7-(4-(4-(Benzyloxy)-2-(3,4-dichloro-5-methyl-1*H*-pyrrole-2-carboxamido)benzo[*d*]thiazole-6-carbonyl)piperazin-1-yl)-1-cyclopropyl-6-fluoro-4-oxo-1,4-dihydroquinoline-3-carboxylic acid (3b)**. Synthesized according to General procedure A using 1b (60 mg, 0.162 mmol) as reactant instead of 1a. The crude product was purified with flash column chromatography using dichloromethane/methanol (15:1) as eluent. Yield: 20 mg (20.1%); white solid; mp > 300 °C. ^1^H NMR (400 MHz, DMSO-*d*_6_) δ [ppm] 1.19 (s, 2H, cyclopropyl-CH_2_), 1.28–1.36 (m, 2H, cyclopropyl-CH_2_), 2.26 (s, 3H, pyrr-CH_3_), 3.39 (s, 4H, 2 × piperazine-CH_2_), 3.58–3.94 (m, 5H, 2 × piperazine-CH_2_, CH), 5.32 (s, 2H, CH_2_Bn), 7.19 (s, 1H, Ar-H′-5), 7.29–7.48 (m, 3H, 3 × Ar-H″ (Bn)), 7.48–7.57 (m, 2H, 2 × Ar-H″ (Bn)), 7.61 (d, *J* = 7.4 Hz, 1H, Ar-H-8), 7.74 (s, 1H, Ar-H′-7), 7.95 (d, *J* = 13.1 Hz, 1H, Ar-H-5), 8.68 (s, 1H, Ar-H-2), 12.06 (s, 1H, pyrr-NH/CONH), 12.22 (s, 1H, pyrr-NH/CONH), 15.19 (s, 1H, COOH). IR (ATR) ν [cm^−^¹] = 2915 (NH), 2843 (OH), 1723 (CO), 1658 (CO), 1626, 1531, 1471, 1402, 1331, 1258, 1131, 1029, 902, 743, 617, 551. HRMS (ESI^+^) *m/z* for C_38_H_32_Cl_2_FN_6_O_6_S ([M + H]^+^): calculated 789.1460, found 789.1401, delta: −7.42 ppm. HPLC: *t*_r_ 7.48 min (98.9% at 254 nm).

**7-(4-((*tert*-Butoxycarbonyl)glycyl)piperazin-1-yl)-1-cyclopropyl-6-fluoro-4-oxo-1,4-dihydroquinoline-3-carboxylic acid (5a).** Synthesized according to General procedure A using (*tert*-butoxycarbonyl)glycine (185 mg, 1.06 mmol) as reactant. During isolation, the phases of the mother liquor after the filtration of the precipitated product were separated, where the water phase was extracted with ethyl acetate (2 × 20 mL), and the organic phases were combined and washed with 10% citric acid (2 × 10 mL) and brine (20 mL). The organic phase was dried over Na_2_SO_4_ and filtered, and the solvent was removed in vacuo. The crude product was purified first by crystallization from methanol, where the mother liquor was evaporated, and the residue was purified again by crystallization from a methanol/diethyl ether mixture. All three precipitates were combined to give 148 mg of pure product. Yield: 148 mg (28.7%); pale yellow solid; mp 218–220 °C. ^1^H NMR (400 MHz, DMSO-*d*_6_) δ [ppm] 1.15–1.23 (m, 2H, cyclopropyl-CH_2_), 1.28–1.35 (m, 2H, cyclopropyl-CH_2_), 1.39 (s, 9H, 3 × CH_3_ (*t*-Bu)), 3.28–3.40 (signal is overlapped with the signal for water, 4H, 2 × piperazine-CH_2_), 3.67 (br s, 4H, 2 × piperazine-CH_2_), 3.79–3.89 (m, 3H, CH_2_CO, CH), 6.84 (t, 1H, *J* = 5.6 Hz, NH), 7.59 (d, 1H, *J* = 7.4 Hz, Ar-H-8), 7.95 (d, 1H, *J* = 13.2 Hz, Ar-H-5), 8.68 (s, 1H, Ar-H-2), 15.20 (s, 1H, COOH). IR (ATR) ν [cm^−^¹] = 3406 (NH), 3001 (OH), 1729 (CO), 1702 (CO), 1650 (CO), 1622, 1496, 1468, 1439, 1387, 1341, 1298, 1243, 1217, 1164, 1098, 1054, 1017, 917, 931, 871, 833, 781, 750, 709, 618, 582. MS (ESI^+^) *m/z* = 489.3 ([M + H]^+^). HRMS (ESI^+^) *m/z* for C_24_H_30_FN_4_O_6_ ([M + H]^+^): calculated: 489.2144, found: 489.2130, delta: −2.80 ppm.

**7-(4-(3-((*tert*-Butoxycarbonyl)amino)propanoyl)piperazin-1-yl)-1-cyclopropyl-6-fluoro-4-oxo-1,4-dihydroquinoline-3-carboxylic acid (5b)**. Synthesized according to General procedure A using 3-((*tert*-butoxycarbonyl)amino)propanoic acid (171 mg, 0.91 mmol) as reactant. During isolation, the phases of the mother liquor after filtration of precipitated product were separated, where water phase was extracted with ethyl acetate (2 × 20 mL), and the organic phases were combined and washed with 10% citric acid (2 × 10 mL) and brine (20 mL). The organic phase was dried over Na_2_SO_4_ and filtered, and the solvent was removed in vacuo. The crude product was purified by crystallization from methanol. The two precipitates were combined to give 243 mg of pure product. Yield: 243 mg (53.4%); pale yellow crystals; mp 185–187 °C. ^1^H NMR (400 MHz, DMSO-*d*_6_) δ [ppm] 1.17–1.23 (m, 2H, cyclopropyl-CH_2_), 1.29–1.36 (m, 2H, cyclopropyl-CH_2_), 1.38 (s, 9H, 3 × CH_3_ (*t*-Bu)), 3.18 (q, 2H, *J* = 6.8 Hz, NHCH_2_CH_2_), 3.29–3.42 (signal is overlapped with the signal for water, 6H, 2 × piperazine-CH_2_, NHCH_2_CH_2_), 3.64–3.73 (m, 4H, 2 × piperazine-CH_2_), 3.83 (sept, 1H, *J* = 4.1 Hz, CH), 6.77 (t, 1H, *J* = 5.6 Hz, CONHCH_2_), 7.59 (d, 1H, *J* = 7.5 Hz, Ar-H-8), 7.95 (d, 1H, *J* = 13.2 Hz, Ar-H-5), 8.68 (s, 1H, Ar-H-2), 15.21 (s, 1H, COOH). IR (ATR) ν [cm^−^¹] = 3272 (NH), 2985 (OH), 1732 (CO), 1700 (CO), 1623 (CO), 1503, 1470, 1382, 1343, 1260, 1237, 1169, 1107, 1056, 1020, 966, 933, 876, 833, 803, 780, 747, 699, 666, 636, 564. MS (ESI^+^) *m/z* = 503.3 ([M + H]^+^). HRMS (ESI^+^) *m/z* for C_25_H_32_FN_4_O_6_ ([M + H]^+^): calculated: 503.2300, found: 503.2290, delta: −2.16 ppm.

General procedure B. Synthesis of compounds **6a-b** and **10** (with **6a** as the example). Compound **5a** (171 mg, 0.350 mmol, 1 equiv.) was suspended in 1,4-dioxane (5 mL) and stirred at room temperature for 5 min. Then, 4 M HCl in 1,4-dioxane (6 mL) was added, and the reaction mixture was stirred at room temperature for 3 h. The solvent was removed in vacuo, and the crude product was purified with crystallization from a methanol/diethyl ether mixture, and dried.

**2-(4-(3-Carboxy-1-cyclopropyl-6-fluoro-4-oxo-1,4-dihydroquinolin-7-yl)piperazin-1-yl)-2-oxoethan-1-aminium chloride (6a).** Yield: 95 mg (63.9%); yellow powder; mp 178–180 °C. ^1^H NMR (400 MHz, DMSO-*d*_6_) δ [ppm] 1.17–1.23 (m, 2H, cyclopropyl-CH_2_), 1.27–1.35 (m, 2H, cyclopropyl-CH_2_), 3.22–3.54 (signal is overlapped with the signal for water, 4H, 2 × piperazine-CH_2_), 3.59–3.65 (m, 2H, piperazine-CH_2_), 3.72–3.78 (m, 2H, piperazine-CH_2_), 3.83 (sept, 1H, *J* = 4.0 Hz, CH), 3.92–3.40 (m, 2H, CH_2_NH_3_^+^), 7.59 (d, 1H, *J* = 7.5 Hz, Ar-H-8), 7.97 (d, 1H, *J* = 13.1 Hz, Ar-H-5), 8.10 (s, 3H, NH_3_^+^), 8.69 (s, 1H, Ar-H-2), 15.19 (s, 1H, COOH). IR (ATR) ν [cm^−^¹] = 3395 (NH), 2921 (OH), 2863 (CH), 2631 (CH), 1719 (CO), 1659 (CO), 1624 (CO), 1493, 1452, 1386, 1336, 1304, 1245, 1146, 1056, 1018, 949, 923, 886, 830, 805, 747, 705, 645, 615, 530. MS (ESI^+^) *m/z* = 389.2 ([M + H]^+^). HRMS (ESI^+^) *m/z* for C_19_H_22_FN_4_O_4_ ([M + H]^+^): calculated: 389.1620, found: 389.1612, delta: −1.93 ppm.

**3-(4-(3-Carboxy-1-cyclopropyl-6-fluoro-4-oxo-1,4-dihydroquinolin-7-yl)piperazin-1-yl)-3-oxopropan-1-aminium chloride (6b).** Synthesized according to General procedure B. Yield: 142 mg (77.1%); pale yellow powder; mp 207–209 °C. ^1^H NMR (400 MHz, DMSO-*d*_6_) δ [ppm] 1.16–1.24 (m, 2H, cyclopropyl-CH_2_), 1.28–1.35 (m, 2H, cyclopropyl-CH_2_), 2.75 (t, 2H, *J* = 6.4 Hz, NH_3_^+^CH_2_CH_2_), 2.98–3.10 (m, 2H, NH_3_^+^CH_2_CH_2_), 3.29–3.50 (signal is overlapped with the signal for water, 4H, 2 × piperazine-CH_2_), 3.63–3.70 (m, 2H, piperazine-CH_2_), 3.70–3.76 (m, 2H, piperazine-CH_2_), 3.83 (sept, 1H, *J* = 4.0 Hz, CH), 7.58 (d, 1H, *J* = 7.5 Hz, Ar-H-8), 7.77 (br s, 3H, NH_3_+), 7.96 (d, 1H, *J* = 13.2 Hz, Ar-H-5), 8.69 (s, 1H, Ar-H-2), 15.19 (s, 1H, COOH). IR (ATR) ν [cm^−^¹] = 3459 (NH), 3233 (NH), 3058 (OH), 2953, 2849 (CH), 1700 (CO), 1621 (CO), 1477, 1443, 1332, 1246, 1216, 1188, 1143, 1107, 1044, 1023, 988, 947, 930, 873, 833, 808, 750, 626, 539. MS (ESI^+^) *m/z* = 403.1 ([M + H]^+^). HRMS (ESI^+^) *m/z* for C_20_H_24_FN_4_O_4_ ([M + H]^+^): calculated: 403.1776, found: 403.1769, delta: −1.71 ppm.

**1-Cyclopropyl-7-(4-((2-(3,4-dichloro-5-methyl-1*H*-pyrrole-2-carboxamido)benzo[*d*]thiazole-6-carbonyl)glycyl)piperazin-1-yl)-6-fluoro-4-oxo-1,4-dihydroquinoline-3-carboxylic acid (7a)**. Synthesized according to General procedure A with 1a (61 mg, 0.165 mmol) and 6a (70 mg, 0.165 mmol) as reactants and 3 equiv. of *N*-methylmorpholine (54 µL, 0.495 mmol) instead of 2 equiv. The precipitate after filtration was resuspended in methanol, sonicated, heated, and filtered off. The procedure was repeated twice. Yield: 29.5 mg (24.2%); light grey powder; mp > 300 °C. ^1^H NMR (400 MHz, DMSO-*d*_6_) δ [ppm] 1.16–1.23 (m, 2H, cyclopropyl-CH_2_), 1.28–1.36 (m, 2H, cyclopropyl-CH_2_), 2.28 (s, 3H, pyrr-CH_3_), 3.42 (s, 4H, 2 × piperazine-CH_2_), 3.69–3.78 (m, 4H, 2 × piperazine-CH_2_), 3.84 (s, 1H, CH), 4.25 (d, 2H, *J* = 5.4 Hz, NHCH_2_CO), 7.61 (d, 1H, *J* = 7.4 Hz, Ar-H′-4/Ar-H-8), 7.82 (s, 1H, Ar-H), 7.93–8.02 (m, 2H, 2 × Ar-H), 8.53 (s, 1H, Ar-H), 8.68 (s, 1H, Ar-H-2), 8.69–8.72 (m, 1H, ArCONH), 11.93 (s, 1H, pyrr-NH/pyrr-CONH), 12.35 (s, 1H, pyrr-NH/pyrr-CONH), 15.21 (s, 1H, COOH). IR (ATR) ν [cm^−^¹] = 3361 (NH), 3232 (NH), 2989 (OH), 1712 (CO), 1660 (CO), 1625 (CO), 1504, 1449, 1331, 1253, 1024, 938, 888, 831, 765, 746, 701, 619. HRMS (ESI^+^) *m/z* for C_33_H_29_Cl_2_FN_7_O_6_S ([M + H]^+^): calculated: 740.1256, found: 740.1226, delta: −3.98 ppm. HPLC: *t*_r_ 6.44 min (95.0% at 254 nm).

**1-Cyclopropyl-7-(4-(3-(2-(3,4-dichloro-5-methyl-1*H*-pyrrole-2-carboxamido)benzo[*d*]thiazole-6-carboxamido)propanoyl)piperazin-1-yl)-6-fluoro-4-oxo-1,4-dihydroquinoline-3-carboxylic acid (7b)**. Synthesized according to General procedure A with 1a (50 mg, 0.135 mmol) and 6b (59 mg, 0.135 mmol) as reactants and 3 equiv. of *N*-methylmorpholine (45 µL, 0.405 mmol) instead of 2 equiv. The precipitate after filtration was suspended in water, sonicated, and filtered off, washed with hot tetrahydrofuran and dried. Yield: 63 mg (61.5%); brown solid; mp > 300 °C. ^1^H NMR (400 MHz, DMSO-*d*_6_) δ [ppm] 1.14–1.22 (m, 2H, cyclopropyl-CH_2_), 1.24–1.36 (m, 2H, cyclopropyl-CH_2_), 2.28 (s, 3H, pyrr-CH_3_), 2.72 (t, 2H, *J* = 7.1 Hz, NHCH_2_CH_2_CO), 3.32 (signal is overlapped with the signal for water, 4H, 2 × piperazine-CH_2_), 3.53–3.58 (m, 2H, NHCH_2_CH_2_ CO), 3.72 (s, 4H, 2 × piperazine-CH_2_), 3.80 (s, 1H, CH), 7.54 (d, 1H, *J* = 7.4 Hz, Ar-H′-4/Ar-H-8), 7.79 (s, 1H, Ar-H), 7.89–7.99 (m, 2H, 2 × Ar-H), 8.48 (s, 1H, Ar-H), 8.63 (t, 1H, *J* = 4.0 Hz, ArCONH), 8.67 (s, 1H, Ar-H-2), 11.90 (s, 1H, pyrr-NH/pyrr-CONH), 12.33 (s, 1H, pyrr-NH/pyrr-CONH), 15.20 (s, 1H, COOH). IR (ATR) ν [cm^−^¹] = 3382 (NH), 3205 (NH), 3002 (OH), 1721 (CO), 1625 (CO), 1508, 1448, 1408, 1372, 1260, 1089, 1025, 887, 807, 767, 743, 699, 621, 573, 521. HRMS (ESI^+^) *m/z* for C_34_H_31_Cl_2_FN_7_O_6_S ([M + H]^+^): calculated: 754.1412, found: 754.1386, delta: −3.53 ppm. HPLC: *t*_r_ 6.25 min (95.1% at 254 nm).

**7-(4-(2-(2-((*tert*-Butoxycarbonyl)amino)ethoxy)ethyl)piperazin-1-yl)-1-cyclopropyl-6-fluoro-4-oxo-1,4-dihydroquinoline-3-carboxylic acid (9)**. To a solution of ciprofloxacin (2, 500 mg, 1.51 mmol) in DMF (33 mL), Et_3_N (694 µL, 4.98 mmol) and 2-(2-((*tert*-butoxycarbonyl)amino)ethoxy)ethyl methanesulfonate (855 mg, 3.02 mmol) dissolved in 3 mL of DMF were added, and the reaction mixture was stirred at 80 °C. Then, 5 drops of water were added, and the reaction mixture was stirred at 80 °C for 2 days. An additional 1 equiv. of 2-(2-((*tert*-butoxycarbonyl)amino)ethoxy)ethyl methanesulfonate (428 mg, 1.51 mmol) dissolved in 3 mL of DMF and 5 drops of water were added, and the reaction mixture was stirred at 80 °C overnight. The solvent was removed in vacuo, and ethyl acetate (200 mL) and water (100 mL) were added to the residue. The phases were separated, with the organic phase dried over Na_2_SO_4_ and filtered, and the solvent removed in vacuo. The crude product was purified with crystallization from methanol, and then additionally purified by addition of water (25 mL), with sonication and filtration of the product. The procedure was repeated twice. Yield: 243 mg (31.0%); pale yellow solid; mp 147–150 °C. ^1^H NMR (400 MHz, DMSO-*d*_6_) δ [ppm] 1.15–1.23 (m, 2H, cyclopropyl-CH_2_), 1.29–1.35 (m, 2H, cyclopropyl-CH_2_), 1.38 (s, 9H, 3 × CH_3_ (*t*-Bu)), 2.57 (t, 2H, *J* = 5.6 Hz, OCH_2_CH_2_N), 2.66-2.69 (m, 4H, 2 × piperazine-CH_2_), 3.09 (q, 2H, *J* = 5.6 Hz, NHCH_2_CH_2_O), 3.30–3.43 (signal is overlapped with the signal for water, 6H, 2 × piperazine-CH_2_, OCH_2_CH_2_N), 3.56 (t, 2H, *J* = 5.6 Hz, NHCH_2_CH_2_O), 3.83 (sept, 1H, *J* = 3.6 Hz, CH), 8.82 (t, 1H, *J* = 5.5 Hz, *t*-BuOCONH), 7.57 (d, 1H, *J* = 7.5 Hz, Ar-H-8), 7.92 (d, 1H, *J* = 13.4 Hz, Ar-H-5), 8.67 (s, 1H, Ar-H-2), 15.25 (s, 1H, COOH). IR (ATR) ν [cm^−^¹] = 3231 (NH), 2969 (OH), 1727 (CO), 1687 (CO), 1628 (CO), 1503, 1451, 1388, 1359, 1338, 1268, 1249, 1166, 1121, 1041, 1027, 1001, 942, 891, 868, 830, 802, 746, 696, 637, 577, 549. MS (ESI^+^) *m/z* = 519.4 ([M + H]^+^). HRMS (ESI^+^) *m/z* for C_26_H_36_FN_4_O_6_ ([M + H]^+^): calculated: 519.2613, found: 519.2606, delta: −1.48 ppm.

**1-(2-(2-Ammonioethoxy)ethyl)-4-(3-carboxy-1-cyclopropyl-6-fluoro-4-oxo-1,4-dihydroquinolin-7-yl)piperazin-1-ium chloride (10).** Synthesized according to General procedure B. Instead of removing the solvent in vacuo, the formed precipitate was filtered off, washed with 1,4-dioxane (10 mL) and dried. Yield: 230 mg (100.0%); grey powder; mp 176–180 °C. ^1^H NMR (400 MHz, DMSO-*d*_6_) δ [ppm] 1.13–1.26 (m, 2H, cyclopropyl-CH_2_), 1.28–1.40 (m, 2H, cyclopropyl-CH_2_), 2.97–3.10 (m, 2H, CH_2_), 3.32–3.92 (signal is overlapped with the signal for water, 15H, 7 × CH_2_, CH), 7.63 (d, 1H, *J* = 7.6 Hz, Ar-H-8), 7.99 (d, 1H, *J* = 13.1 Hz, Ar-H-5), 8.25 (s, 3H, NH_3_^+^), 8.71 (s, 1H, Ar-H-2), 11.12 (s, 1H, NH^+^), signal for COOH group is not seen. IR (ATR) ν [cm^−^¹] = 3422 (NH), 3343 (NH), 2971 (OH), 1717 (CO), 1628 (CO), 1612, 1505, 1485, 1453, 1431, 1376, 1335, 1278, 1111, 1066, 1014, 943, 902, 884, 809, 786, 704, 627, 549. MS (ESI^+^) *m/z* = 419.2 ([M + H]^+^). HRMS (ESI^+^) *m/z* for C_21_H_28_FN_4_O_4_ ([M + H]^+^): calculated: 419.2089, found: 419.2082, delta: −1.65 ppm.

**4-(3-Carboxy-1-cyclopropyl-6-fluoro-4-oxo-1,4-dihydroquinolin-7-yl)-1-(2-(2-(2-(3,4-dichloro-5-methyl-1*H*-pyrrole-2-carboxamido)benzo[*d*]thiazole-6-carboxamido)ethoxy)ethyl)piperazin-1-ium chloride (11a)**. Synthesized according to General procedure A with 1a (69 mg, 0.186 mmol) and 10 (92 mg, 0.186 mmol) as reactants. After isolation the product was additionally purified by crystallization from methanol. Yield: 36 mg (25.3%); light purple solid; mp 187–190 °C. ^1^H NMR (400 MHz, DMSO-*d*_6_) δ [ppm] 1.20–1.27 (m, 2H, cyclopropyl-CH_2_), 1.28–1.35 (m, 2H, cyclopropyl-CH_2_), 2.28 (s, 3H, pyrr-CH_3_), 3.12–3.31 (m, 4H, 2 × CH_2_), 3.39–3.84 (s, 4H, 2 × CH_2_) 3.52–3.61 (m, 4H, 2 × CH_2_), 3.67 (t, 2H, *J* = 4.8 Hz, CH_2_), 3.78–3.92 (m, 3H, CH_2_, CH), 7.39 (d, 1H, *J* = 7.4 Hz, Ar-H), 7.70–7.83 (m, 2H, 2 × Ar-H), 7.98 (dd, 1H, *J* = 1.7, 8.5 Hz, Ar-H), 8.57 (s, 2H, 2 × Ar-H), 8.70 (t, 1H, *J* = 4.9 Hz, ArCONH), 10.73 (s, 1H, NH^+^), 11.68 (s, 1H, pyrr-NH/pyrr-CONH), 12.23 (s, 1H, pyrr-NH/pyrr-CONH), 14.91 (s, 1H, COOH). IR (ATR) ν [cm^−^¹] = 3365 (NH), 3223 (NH), 2925 (OH), 1719 (CO), 1657 (CO), 1627 (CO), 1525, 1455, 1400, 1331, 1264, 1167, 1104, 1036, 944, 913, 892, 833, 748, 702, 621, 551, 519. HRMS (ESI^+^) *m/z* for C_35_H_35_Cl_2_FN_7_O_6_S ([M + H]^+^): calculated: 770.1725, found: 770.1725, delta: −0.03 ppm. HPLC: *t*_r_ 4.82 min (95.1% at 254 nm).

**1-(2-(2-(4-(Benzyloxy)-2-(3,4-dichloro-5-methyl-1*H*-pyrrole-2-carboxamido)benzo[*d*]thiazole-6-carboxamido)ethoxy)ethyl)-4-(3-carboxy-1-cyclopropyl-6-fluoro-4-oxo-1,4-dihydroquinolin-7-yl)piperazin-1-ium chloride (11b)**. Synthesized according to General procedure A with 1b (40 mg, 0.084 mmol) and 10 (41 mg, 0.084 mmol) as reactants. After isolation, the product was additionally purified by washing with hot water three times and by crystallization from methanol. Yield: 3.42 mg (4.5%); light beige solid; mp > 300 °C. ^1^H NMR (400 MHz, DMSO-*d*_6_) δ [ppm] 1.13–1.22 (m, 4H, 2 × cyclopropyl-CH_2_), 2.25 (s, 3H, pyrr-CH_3_), 3.03 (t, 2H, *J* = 12.1 Hz, CH_2_), 3.24–3.29 (m, 2H, CH_2_), 3.39–3.48 (m, 4H, 2 × CH_2_), 3.52–3.61 (m, 4H, 2 × CH_2_), 3.63–3.70 (m, 2H, CH_2_), 3.74–3.79 (m, 1H, CH), 3.84 (s, 2H, CH_2_), 5.29 (s, 2H, Bn-CH_2_), 7.26 (d, 1H, *J* = 7.2 Hz, Ar-H), 7.35–7.47 (m, 3H, 3 × Ar-H), 7.48–7.55 (m, 2H, 2 × Ar-H), 7.64 (d, *J* = 1.5 Hz, Ar-H), 7.75 (d, 1H, *J* = 12.9 Hz, Ar-H), 8.12 (s, 1H, Ar-H), 8.54 (s, 1H, Ar-H), 8.64 (s, 1H, ArCONH), 10.12 (s, 1H, NH^+^), 11.80 (s, 1H, pyrr-NH/pyrr-CONH), 11.92 (s, 1H, pyrr-NH/pyrr-CONH), 14.87 (s, 1H, COOH). HRMS (ESI^+^) *m/z* for C_42_H_41_Cl_2_FN_7_O_7_S ([M + H]^+^): calculated: 876.2144, found: 876.2135, delta: −1.00 ppm. HPLC: *t*_r_ 5.54 min (93.1% at 254 nm).
