# Supplementary material for: Hybrid Inhibitors of DNA Gyrase A and B: Design, Synthesis and Evaluation
Source: Pharmaceutics. 2020 Dec 22;13(1):6. doi: 10.3390/pharmaceutics13010006 (PMC7822030; doi:10.3390/pharmaceutics13010006)
Supplement: Supplementary file 1 [file pharmaceutics-13-00006-s001.pdf]

pharmaceutics

MDPI

Supplementary Materials: Hybrid Inhibitors of DNA Gyrase A and B: Design, Synthesis and Evaluation

Martina Durcik, Žiga Skok, Janez Ilaš, Nace Zidar, Anamarija Zega, Petra Éva Szili, Gábor Draskovits, Tamás Révész, Danijel Kikelj, Akos Nyerges, Csaba Pál, Lucija Peterlin Mašič and Tihomir Tomašič

1. Enzyme Inhibition Graphs

Dose-response curves for six hybrids active against *E. coli* DNA gyrase in supercoiling assay (Figure S1) and for four hybrids active against *E. coli* topoisomerase IV in relaxation assay (Figure S2), shown for an independent measurement. The IC_{50} (mean ±SD) is the result of at least two independent measurements.

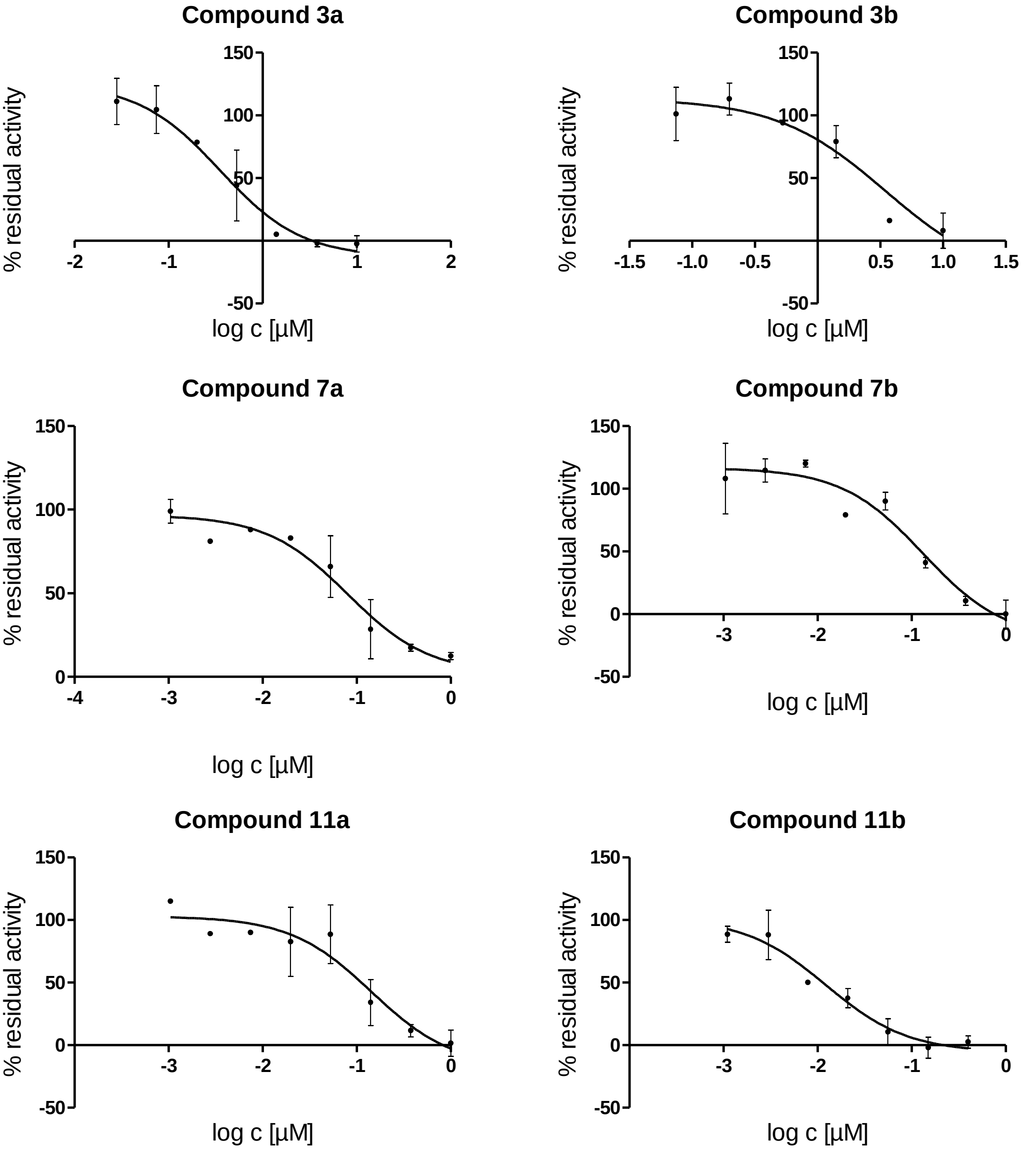

Figure S1. Dose-response curves for compounds **3a**, **3b**, **7a**, **7b**, **11a**, and **11b** for *E. coli* DNA gyrase.

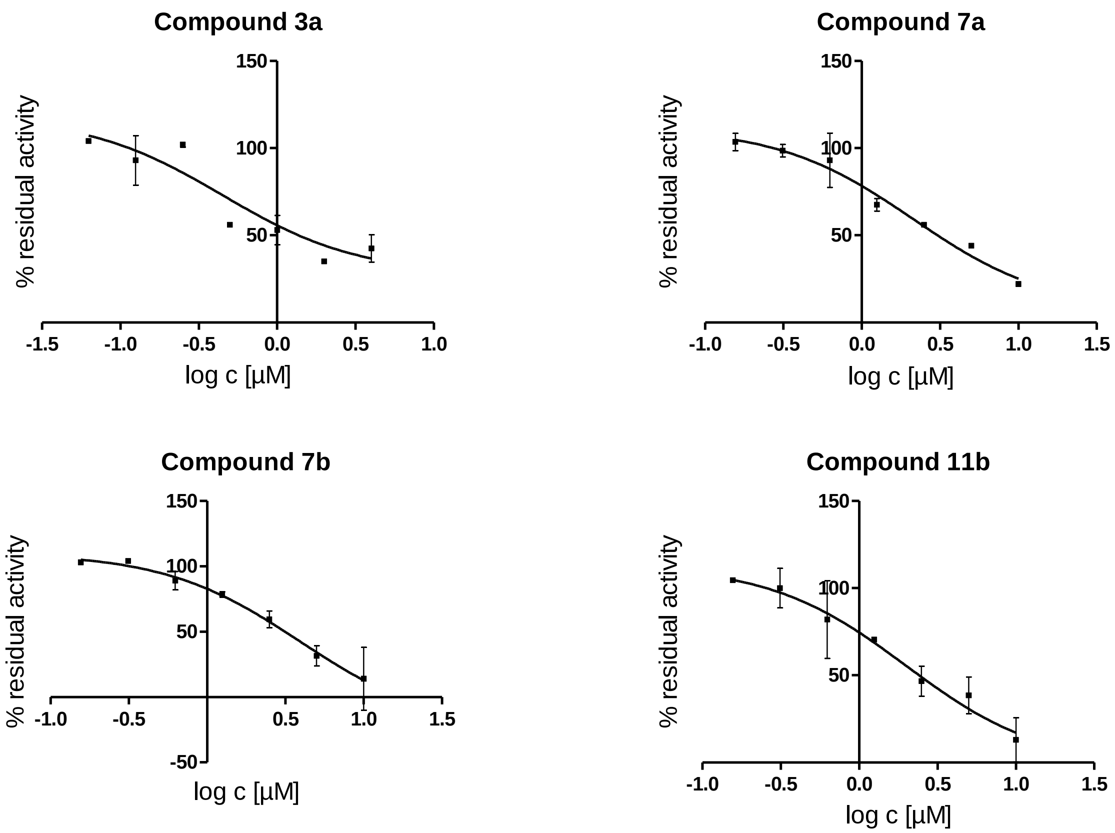

Figure S2. Dose-response curves for compounds **3a**, **7a**, **7b**, and **11b** for *E. coli* topoisomerase IV.

2. ^{1}H NMR Spectra and HPLC Chromatograms of Final Compounds

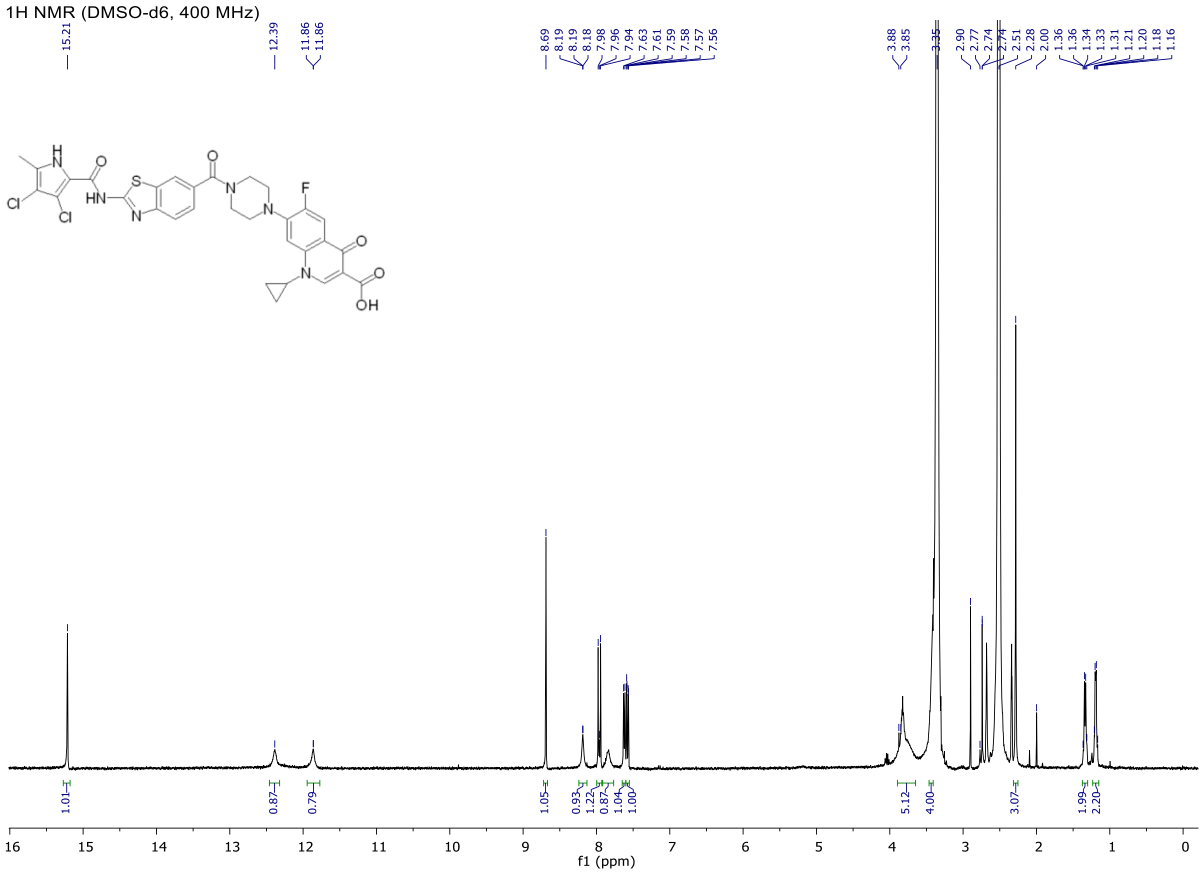

Figure S3. ^{1}H NMR spectra of compound **3a**.

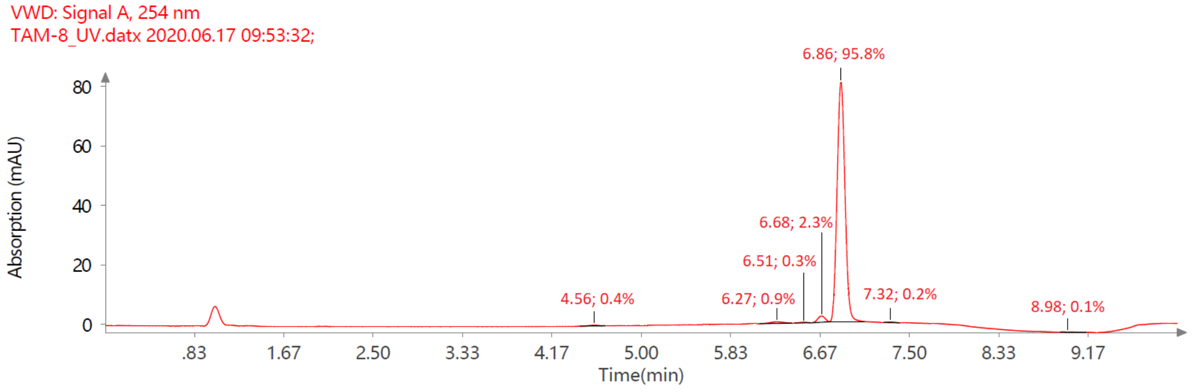

Figure S4. HPLC chromatogram of compound **3a**.

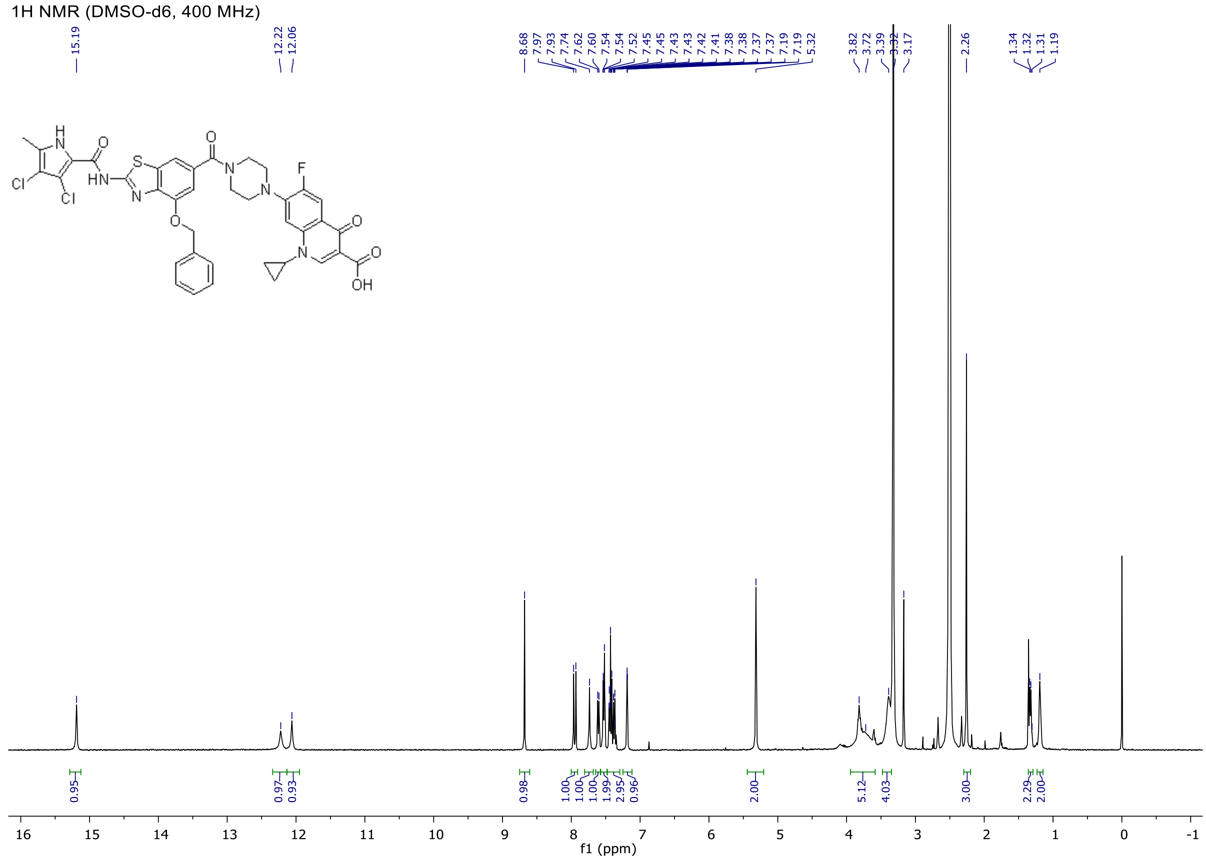

Figure S5. ^{1}H NMR spectra of compound **3b**.

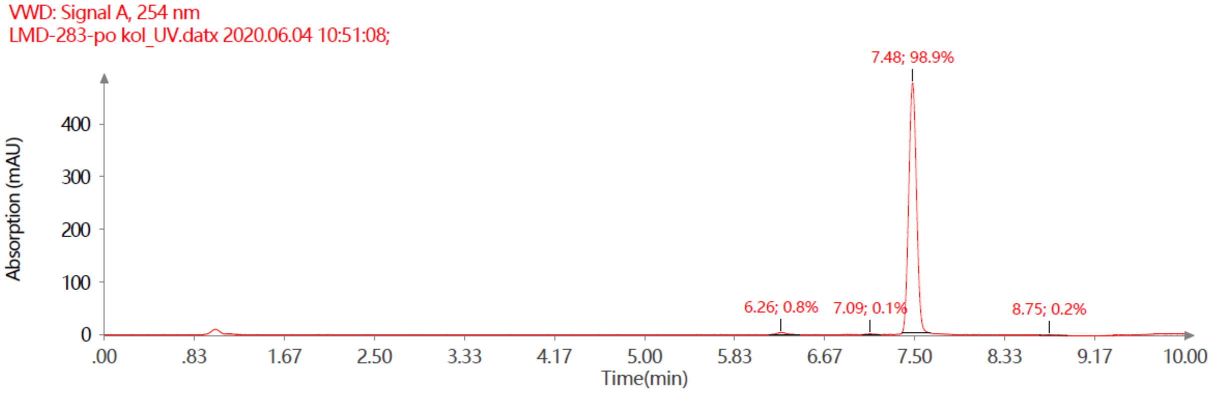

Figure S6. HPLC chromatogram of compound **3b**.

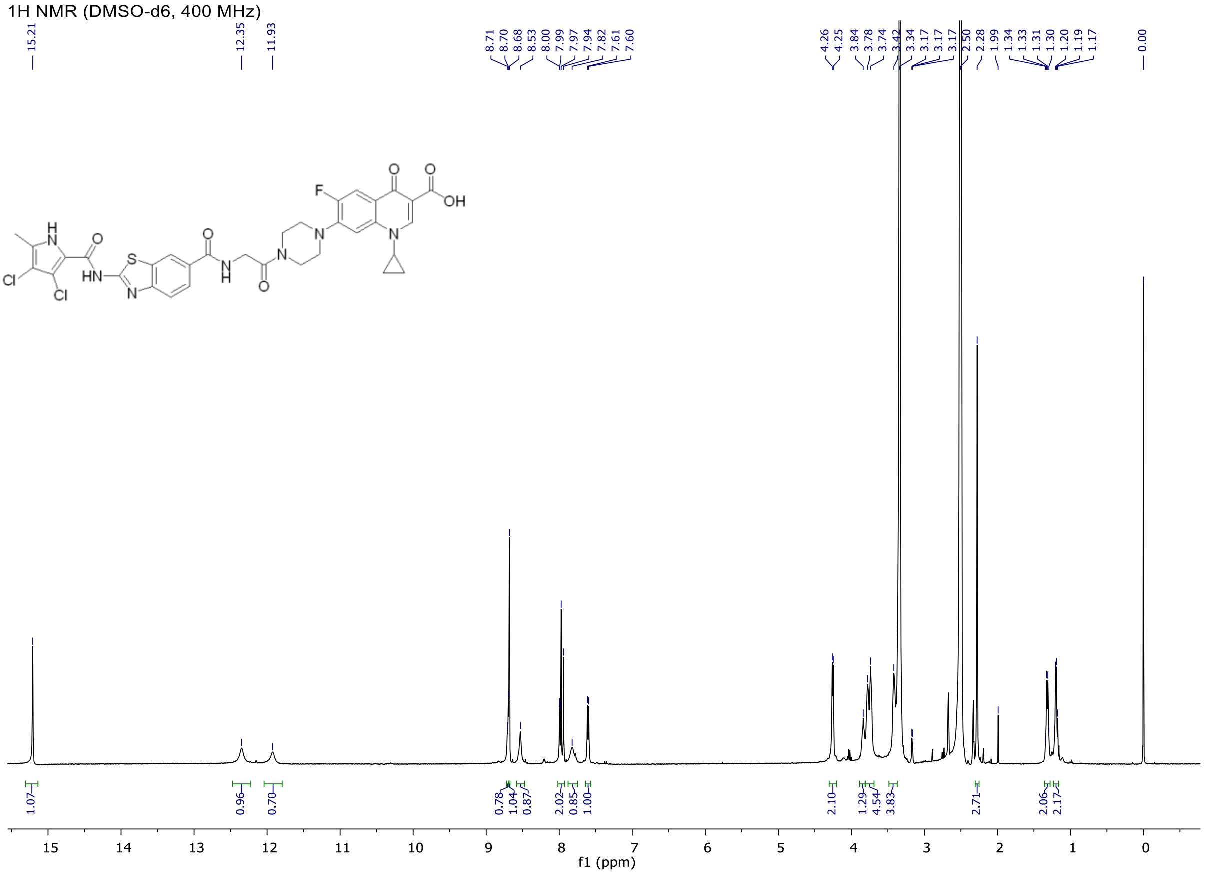

Figure S7. ¹H NMR spectra of compound **7a**.

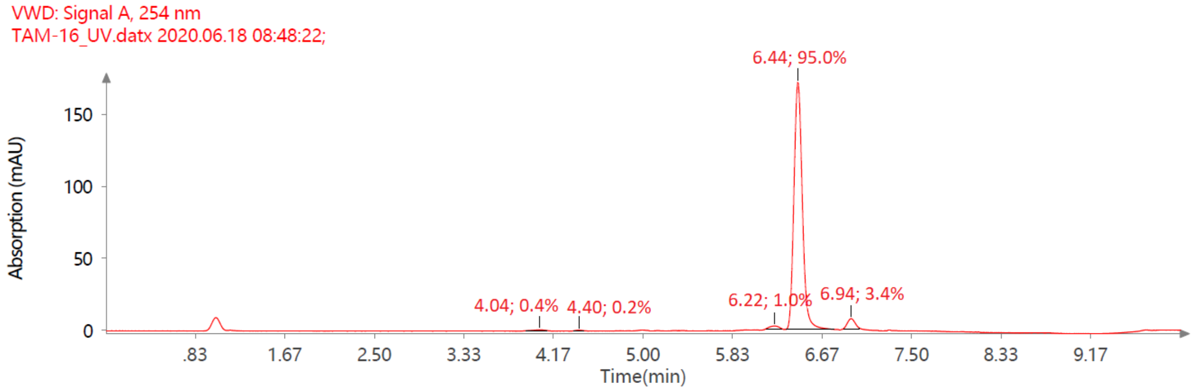

Figure S8. HPLC chromatogram of compound **7a**.

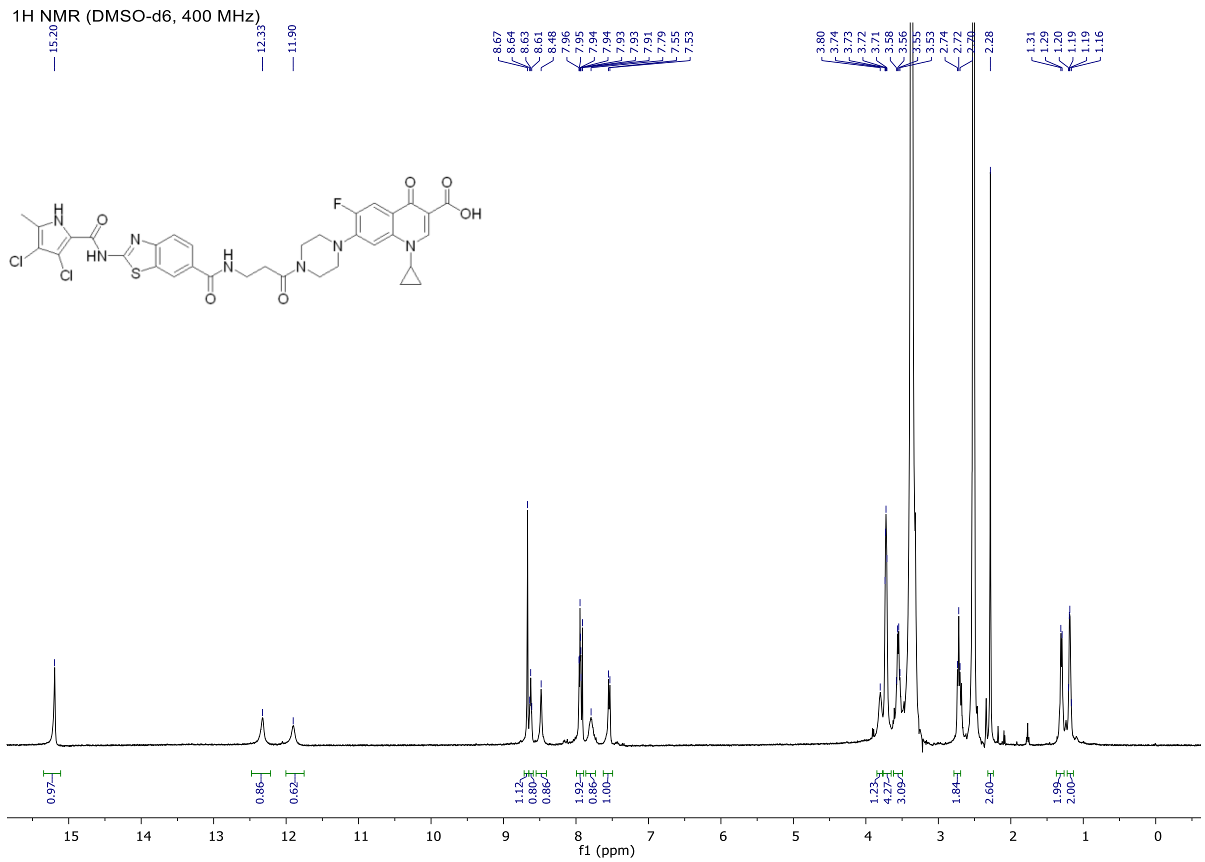

Figure S9. ^{1}H NMR spectra of compound **7b**.

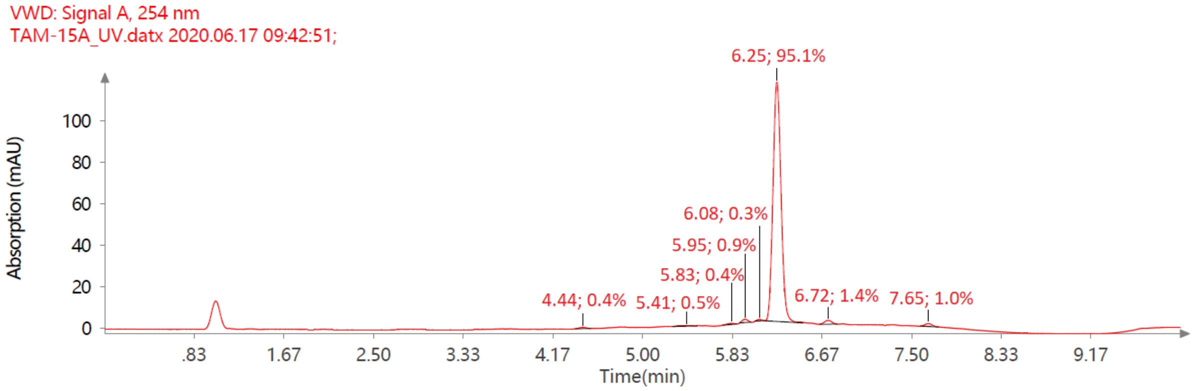

Figure S10. HPLC chromatogram of compound **7b**.

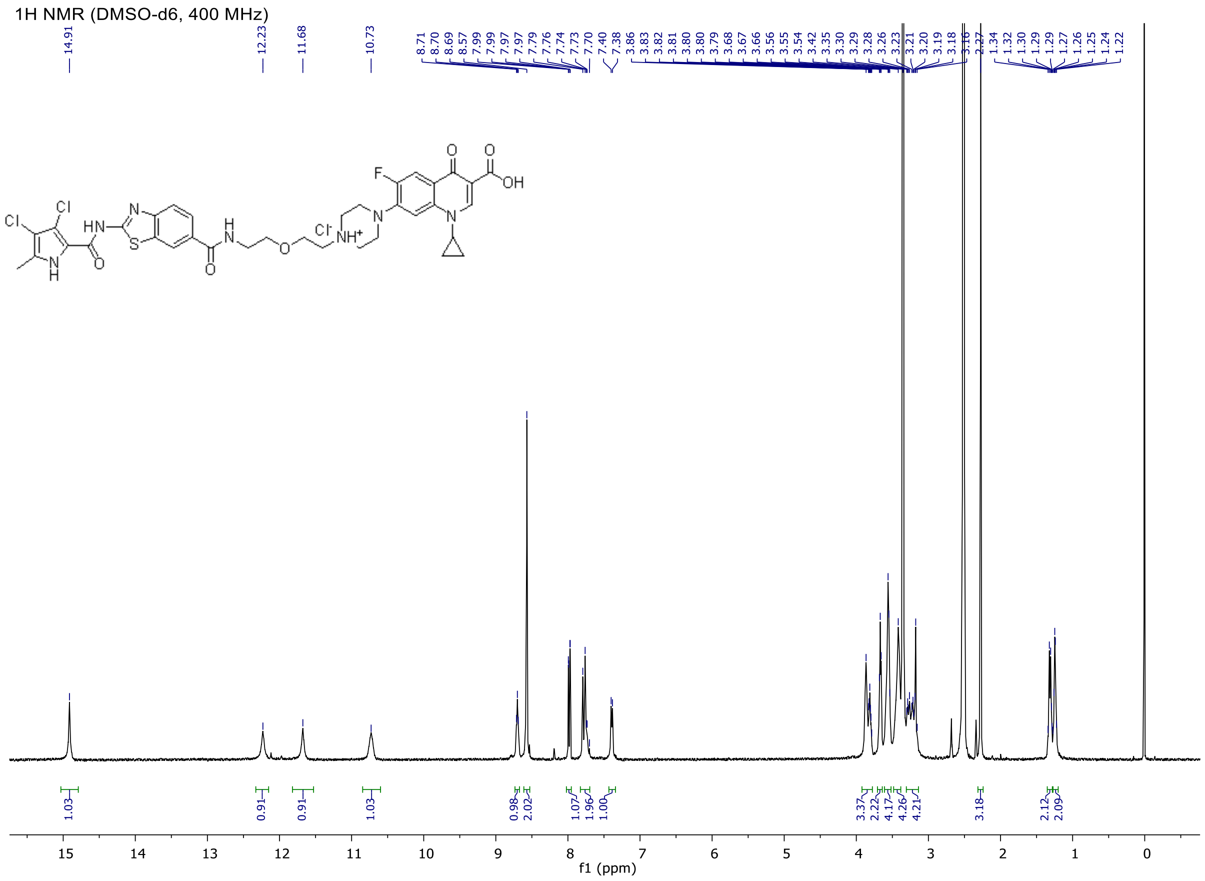

Figure S11. ^{1}H NMR spectra of compound **11a**.

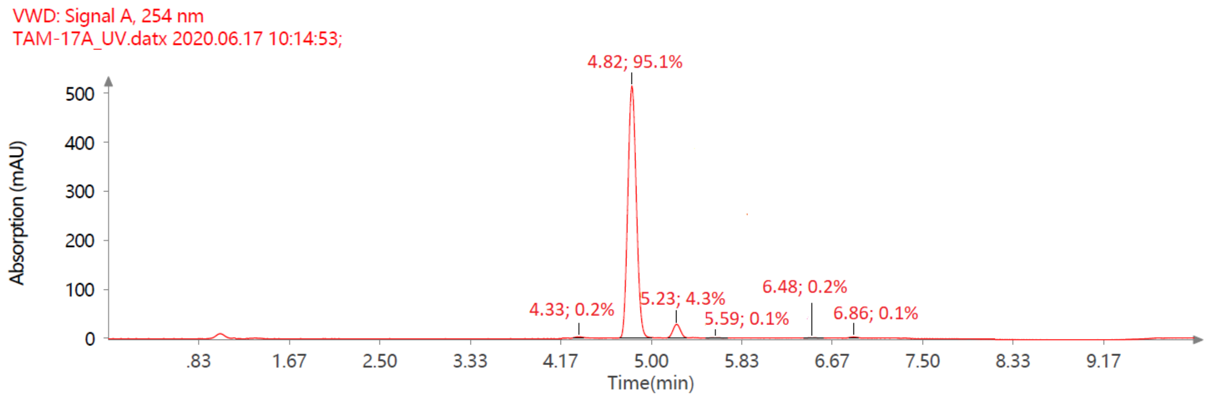

Figure S12. HPLC chromatogram of compound **11a**.

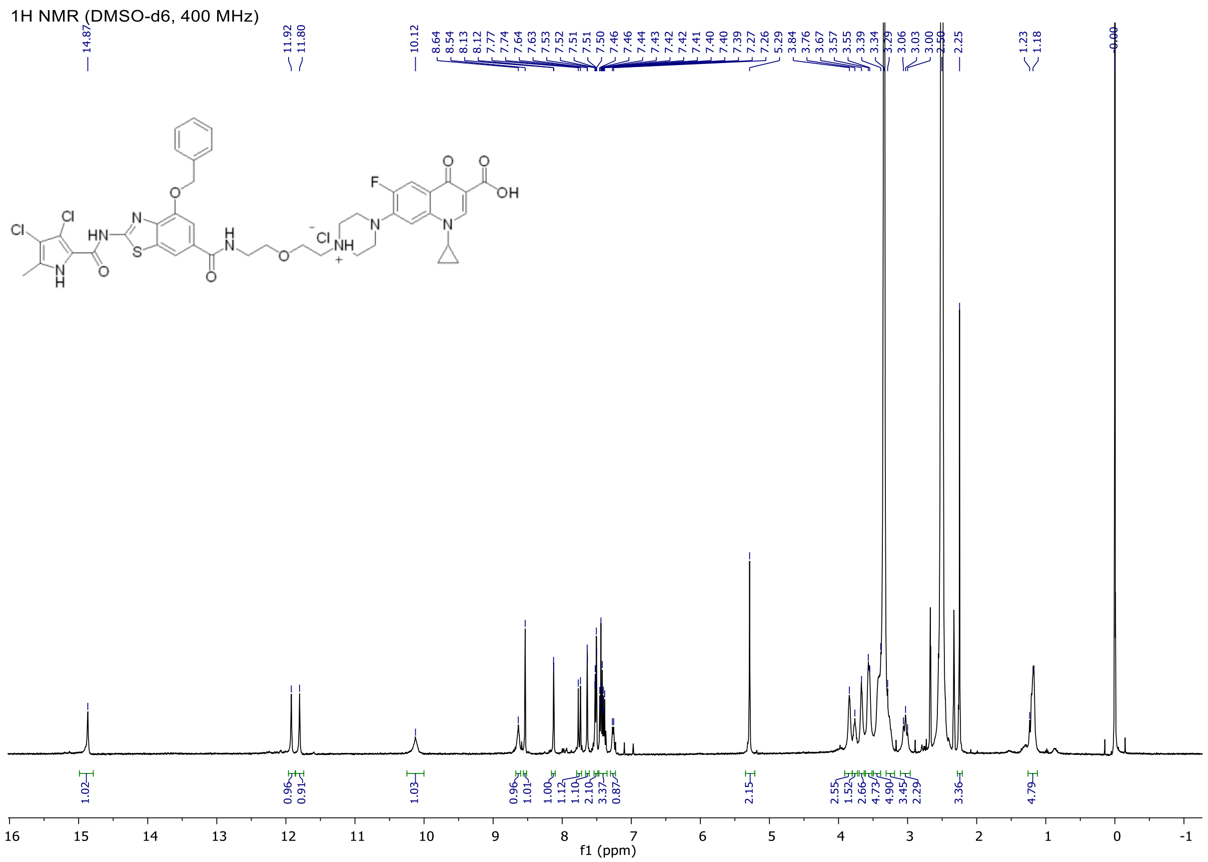

Figure S13. ^{1}H NMR spectra of compound **11b**.

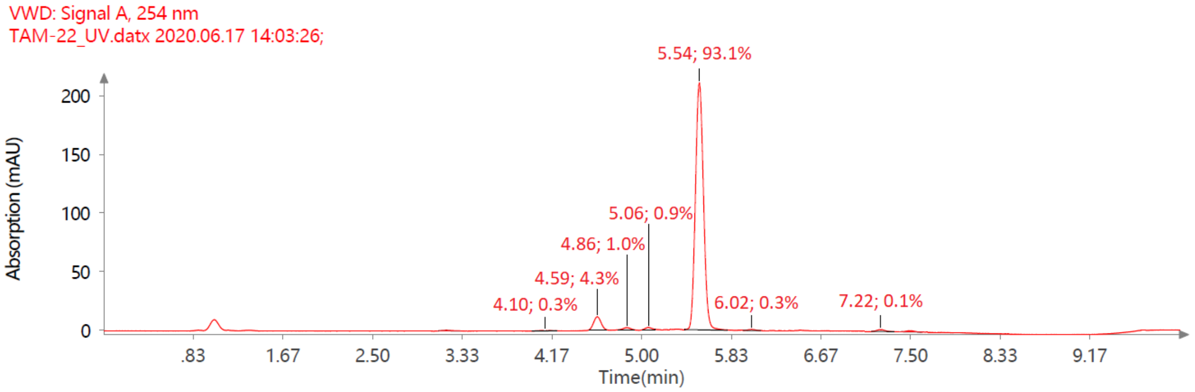

Figure S14. HPLC chromatogram of compound **11b**.
